# Supplementary material for: Experience of induction of labour: a cross-sectional postnatal survey of women at UK maternity units
Source: BMJ Open. 2023 May 9;13(5):e071703. doi: 10.1136/bmjopen-2023-071703 (PMC10174017; doi:10.1136/bmjopen-2023-071703)
Supplement: Author's manuscript [file bmjopen-2023-071703.draft_revisions.pdf]

BMJ Open is committed to open peer review. As part of this commitment we make the peer review history of every article we publish publicly available.

When an article is published we post the peer reviewers' comments and the authors' responses online. We also post the versions of the paper that were used during peer review. These are the versions that the peer review comments apply to.

The versions of the paper that follow are the versions that were submitted during the peer review process. They are not the versions of record or the final published versions. They should not be cited or distributed as the published version of this manuscript.

BMJ Open is an open access journal and the full, final, typeset and author-corrected version of record of the manuscript is available on our site with no access controls, subscription charges or pay-per-view fees (http://bmjopen.bmj.com).

If you have any questions on BMJ Open's open peer review process please email info.bmjopen@bmj.com

BMJ Open

Experience of induction of labour: a cross-sectional postnatal survey of women at UK maternity units

Journal:	*BMJ Open*
Manuscript ID	bmjopen-2023-071703
Article Type:	Original research
Date Submitted by the Author:	17-Jan-2023
Complete List of Authors:	Harkness, Mairi; University of Stirling, NMAHP Research Unit<br>Yuill, Cassandra; City University of London, Centre for maternal and child health research<br>Cheyne, Helen; Nursing Midwifery and Allied Health Professions Research Unit, University of Stirling<br>McCourt, Christine; Department of Midwifery and Child health, City University London<br>Black, Mairead; Aberdeen Maternity Hospital, Aberdeen centre for women's health research<br>Pasupathy, Dharmintra; The University of Sydney<br>Sanders, Julia; Cardiff University, School of healthcare sciences<br>Heera, Neelam; Cysters<br>Wallace, Chlorice; Princess Royal University Hospital<br>Stock, Sarah; The University of Edinburgh Usher Institute of Population Health Sciences and Informatics; The University of Edinburgh MRC Centre for Reproductive Health
Keywords:	Decision Making, OBSTETRICS, Quality in health care < HEALTH SERVICES ADMINISTRATION & MANAGEMENT

SCHOLARONE™
Manuscripts

I, the Submitting Author has the right to grant and does grant on behalf of all authors of the Work (as defined in the below author licence), an exclusive licence and/or a non-exclusive licence for contributions from authors who are: i) UK Crown employees; ii) where BMJ has agreed a CC-BY licence shall apply, and/or iii) in accordance with the terms applicable for US Federal Government officers or employees acting as part of their official duties; on a worldwide, perpetual, irrevocable, royalty-free basis to BMJ Publishing Group Ltd ("BMJ") its licensees and where the relevant Journal is co-owned by BMJ to the co-owners of the Journal, to publish the Work in this journal and any other BMJ products and to exploit all rights, as set out in our licence.

The Submitting Author accepts and understands that any supply made under these terms is made by BMJ to the Submitting Author unless you are acting as an employee on behalf of your employer or a postgraduate student of an affiliated institution which is paying any applicable article publishing charge ("APC") for Open Access articles. Where the Submitting Author wishes to make the Work available on an Open Access basis (and intends to pay the relevant APC), the terms of reuse of such Open Access shall be governed by a Creative Commons licence – details of these licences and which Creative Commons licence will apply to this Work are set out in our licence referred to above.

Other than as permitted in any relevant BMJ Author's Self Archiving Policies, I confirm this Work has not been accepted for publication elsewhere, is not being considered for publication elsewhere and does not duplicate material already published. I confirm all authors consent to publication of this Work and authorise the granting of this licence.

For peer review only

Title Page

Title

Experience of induction of labour: a cross-sectional postnatal survey of women at UK maternity units

Authors

Dr Mairi Harkness; Dr Cassandra Yuill; Professor Helen Cheyne; Professor Christine McCourt; Dr Mairead Black; Professor Dharmintra Pasupathy; Professor Julia Sanders; Chloe Wallace; Neelam Heera-Shergill; Professor Sarah Stock

Corresponding Author

Dr Mairi Harkness, NMAHP Research Unit, Pathfoot Building, University of Stirling, Stirling, FK9 4LA. Email: mairi.harkness@stir.ac.uk; Phone: 01786 466341.

Author Details

Dr Mairi Harkness, Nursing Midwifery and Allied Health Professionals Research Unit (NMHAP-RU), University of Stirling, Stirling, UK.

Dr Cassandra Yuill, Centre for Maternal and Child Health Sciences, City, University of London, London, UK.

Professor Helen Cheyne, Nursing Midwifery and Allied Health Professionals Research Unit (NMHAP-RU), University of Stirling, Stirling, UK.

Professor Christine McCourt, Centre for Maternal and Child Health Sciences, City, University of London, London, UK.

Dr Mairead Black, Aberdeen Maternity Hospital, Aberdeen, UK.

Professor Dharmintra Pasupathy, University of Sydney, Sydney, Australia

Professor Julia Sanders, School of Healthcare Sciences, University of Cardiff, Cardiff, UK

Chloe Wallace, Princess Royal University Hospital, Orpington, UK

Neelam Heera-Shergill, Cysters (CIO), Birmingham, UK

Professor Sarah J Stock, Usher Institute, and MRC Centre for Reproductive Health, University of Edinburgh, Edinburgh, UK

Key Words

Pregnant women
Peripartum period
Maternal health services

For peer review only - http://bmjopen.bmj.com/site/about/guidelines.xhtml

Word Count

4106

Footnote: The words woman and women are used throughout this piece however it is important to acknowledge that not all people who have induction of labour identify as women.

Abstract

Objectives: This study explored women's views and experiences of key elements of the induction of labour (IOL) process, including at home or in hospital cervical ripening (CR).

Design: A questionnaire-based postnatal survey undertaken as part of the CHOICE Study process evaluation. The questionnaire was administered online and included fixed response and free text options.

Setting: NHS maternity units in the United Kingdom

Participants: 309 women who had an IOL

Outcome measures: The primary outcome measure was experience of IOL. Few women returned home during CR, meaning that statistical comparison between those who experienced home and hospital based CR was not possible. Findings are reported as descriptive statistics with content analysis of women's comments providing context.

Findings: Information to support choice and understand what to expect about IOL is often inadequate or unavailable. Having IOL can create anxiety and remove options for birth that women had hoped would enhance their experience. Although it can provide a more comfortable environment, home CR is not always an acceptable solution. Women described maternity care negatively impacted by staffing shortages; delays to care sometimes led to unsafe situations. Women who had a positive experience of IOL described supportive interaction with staff as a significant contribution to that.

Conclusions: Women do not experience IOL as a benign and consequence free intervention. There is urgent need for research to better target IOL and optimise safety and experience for women and their babies. Relatively few women were offered CR at home and further research is needed on this experience.

Strengths and Limitations

- A robustly designed survey, including use of previously tested tools, was used to determine key aspects of women's experience of induction of labour.
- Carefully considered recruitment strategies resulted in a large sample across multiple NHS sites.
- Few women returned home during cervical ripening. As a result data analysis produced descriptive, rather than inferential, statistics.
- Qualitative analysis of women's free text comments adds important context and aids understanding and interpretation of the findings.

For peer review only - http://bmjopen.bmj.com/site/about/guidelines.xhtml

- The survey was conducted during the COVID-19 pandemic and findings should be considered within this unique context.

Key Messages

What is already known on this topic

Experience of childbirth is important to women and known to affect options and outcomes; negative experience of childbirth has been linked to serious psychological harm. Despite an IOL rate in the UK of 30-50%, there is a dearth of evidence about how it impacts experience of birth. Home cervical ripening has been suggested as an option that may improve experience, but there is little evidence about its acceptability to women.

What this study adds

This study shows that women do not experience IOL as a benign and consequence free intervention. For many respondents it caused significant anxiety and for some resulted in long term harm. Women reported IOL was often offered on the basis that it was the only safe option, yet the hospital environment and support available during the early stage of the process was frequently inadequate, and sometimes led to physically and emotionally unsafe situations. Inadequate information and staffing shortages were described as underpinning many shortfalls in care. The offer of home CR was not common and experience of this was variable, suggesting that maternal information and choice about home or hospital CR is important.

How this study might affect research, practice or policy

Induction of labour rates have been increased with good intentions of reducing rates of stillbirth and severe neonatal morbidity, but this does not always appear to have been accompanied by adequate planning or increase in the facilities and resources that are necessary to provide an effective service.

Listening to women and service users, ensuring that practice is based on their needs and that genuine informed choice is offered is important for quality care. Services need to be sufficiently resourced to support the provision of safe and effective induction pathways.

Manuscript

Experience of induction of labour: a cross-sectional postnatal survey of women at UK maternity units

For peer review only - http://bmjopen.bmj.com/site/about/guidelines.xhtml

Background

In the UK induction of labour (IOL) rates have increased steadily over the last 20 years with rates currently around 30-50% of births, making IOL one of the most common obstetric interventions (1,2).

IOL is understood to affect experience of childbirth: it is generally more painful than spontaneous labour; more likely to lead to additional interventions such as operative birth and epidural analgesia; and may remove the satisfaction of experiencing the more natural birth that many hope for (3,4).

Women's experience during childbirth is described by the World Health Organisation (WHO) as a 'critical aspect of ensuring high-quality labour and childbirth care (5). Evidence underpins socio-cultural and psychological aspects of care as significant for women during childbirth (6), and negative experience of childbirth can be linked to serious psychological harm (7). Despite this there remains a dearth of evidence about women's experience of IOL.

The first stage of IOL, cervical ripening (CR), involves application of a drug or mechanical method to change a woman's cervix in preparation for labour. The second phase, if labour onset does not occur as a result of CR alone, is artificial rupture of the fetal membranes and intravenous administration of oxytocin. Traditionally the whole process of IOL has been undertaken in hospital, however, some maternity units now offer home CR: women attend hospital for initial assessment and administration of CR agent and then return home for a period before labour starts or reassessment in hospital. Some evidence indicates that home cervical ripening could reduce duration of hospital stay during IOL and improve women's experience, however, its safety and acceptability has not been fully evaluated (8).

The aim of this study was to explore women's views of their IOL, with a specific focus on their experiences of the initial stages of the process including CR at home and in-hospital.

Methods

Design

This study was undertaken as part of the CHOICE Study, a prospective cohort study and process evaluation[8] commissioned by the National Institute for Health and Care Research (NIHR) to examine the safety, efficacy and acceptability of home CR and CR in hospital. The process evaluation (qCHOICE) included a postnatal questionnaire-based survey (reported here).
A cross-sectional online survey was used with the aim of describing women's views and experience of IOL, particularly those having CR at home and in-hospital.

Questionnaire development

The questionnaire was designed to explore key elements of the IOL process, using fixed response and free-text questions. Questions designed to assess satisfaction, sense of control and mental wellbeing were based on previously tested tools (9,10).

A modified version of the IOL satisfaction questionnaire (9) was used to assess women's experiences of IOL, including information provision, anxiety, and physical and emotional discomfort. Responses

For peer review only - http://bmjopen.bmj.com/site/about/guidelines.xhtml

were a five-point Likert scale (Strongly agree – strongly disagree), analysed using N (%) agreement to create three categories: merging strongly agree with agree, and strongly disagree with disagree.

A series of ten questions from the labour agentry scale (short) (10) were used to measure sense of control during childbirth. Responses used a six-point Likert type scale, and analysis was reported as percentage agreement across three categories: agree, neutral and disagree.

Demographic questions and questions about information and decision-making were based on those in the Scottish National Maternity Survey (11) altered to focus on IOL. The survey also included questions about cost of CR (home and hospital), including travel and childcare, for health economics evaluation to be reported elsewhere.

The questionnaire was pilot tested with the CHOICE Study personal and public involvement (PPI) group (women with recent experience of childbirth) and with maternal health researchers at City, University of London. Feedback was used to modify survey questions, particularly those concerning decision-making and choice, improving clarity and accessibility.

The survey was online, hosted by *Online Surveys* (www.onlinesurveys.ac.uk); a written version or completion via telephone and/or with a translator could be requested.

Sample and recruitment

A convenience sampling approach was used, with women who underwent IOL at 37 weeks of pregnancy or more at the 21 NHS sites participating in the CHOICE study potentially eligible. Those who experienced pregnancy loss were ineligible. A sample size of 300 respondents was deemed practical, achievable and useful for the purpose of describing the experience of women who undergo CR.

The initial recruitment strategy was via electronic maternity notes system BadgerNet. We anticipated that women would receive information about the study using push notifications sent when an IOL was booked, and again at around ten days postnatal when maternity care ended; directing them to view study information on their electronic maternity notes and access the study link. However, initial response rate was poor, it was not clear to what extent push notifications were being received, therefore additional strategies were put in place while the survey remained open: firstly, efforts were targeted to seven sites (the five case study sites plus two that had expressed interest in the survey) to obtain a more focused response. At these sites a research midwife identified eligible participants on the postnatal ward and handed them a study card with a link to the online survey; In addition, a targeted social media advertising campaign (Facebook) was used in the five qCHOICE case study sites.

The survey was open between February 2021 and April 2022.

Patient and public involvement statement

The CHOICE Study PPI group were involved in development of participant information materials and survey recruitment strategies. The survey questionnaire was pilot tested with the group. A member of the PPI group is co-author for this paper and will be involved in further dissemination of findings.

For peer review only - http://bmjopen.bmj.com/site/about/guidelines.xhtml

Consent for participation

The questionnaire landing page included detailed participant information, researcher contact details for further information, and consent questions to be completed before the survey could be accessed.

Data Analysis

Survey responses were exported from the online survey site into IBM SPSS Statistics 23 software. Data were de-identified, cleaned and statistics produced.

We found that 12% (n=36) of the eligible survey respondents returned home during CR therefore it was not possible to use inferential statistics to compare their experience with those who remained in hospital. Instead, descriptive analysis was used across both groups with analysis of free text responses providing context to the women's experience.

Free-text responses were analysed using thematic analysis approach; determining themes and, for some questions, how often those themes occurred. NVivo 12 software was used to organise the data and assist analysis.

Findings

In total 320 questionnaires were completed. Nine responses were excluded as respondents had not had an IOL and a further two because their IOL happened prior to the CHOICE Study commencing. Three hundred and nine eligible responses were included in the analysis.

Study Respondents

Respondents had given birth in Scotland, England and Wales at 19 CHOICE Study sites and a further six NHS areas. Descriptive data for those who responded are given in Table 1.

For peer review only

For peer review only - http://bmjopen.bmj.com/site/about/guidelines.xhtml

Table 1: Description of the survey respondents

First baby?<br>N=309	Yes:<br>206 (67%)	No:<br>103 (33%)			
Maternal Age (years)<br>N=309	Min: 19	Max: 52	Median: 31		
Ethnicity<br>N=307<br>(2 missing)	White<br><br>291 (95%)	Asian/Asian British<br><br>8 (3%)	Black:<br><br>4 (1%)	Mixed/multiple ethnicity:<br><br>4 (1%)	
Social deprivation index<br>N=306 (3 missing)	1<br>(most deprived)<br><br>61 (20%)	2<br><br>57 (19%)	3<br><br>60 (20%)	4<br><br>73 (24%)	4<br>(least deprived)<br><br>55 (18%)
Baby's birthweight (grams)<br>N=297<br>(12 missing)	Min: 1790	Max: 6600	Median: 3500		
Gestation at IOL (weeks)<br>N=309	Min:37	Max: 42	Median: 39		
Reason for IOL<br>N=309	Medical<br>(eg raised blood pressure)<br><br>146 (47%)	Post dates<br><br>70 (23%)	Size of baby<br>(large or small)<br><br>37 (12%)	Spontaneous rupture of membranes (SROM)<br>20 (7%)	Reduced fetal movements (RFM)<br><br>19 (6%)
Cervical ripening?<br>N=304<br>(5 missing)	Yes<br><br>266 (86%)	No<br><br>38 (12%)			
Method of induction of labour<br>N=309	Prostaglandin gel/pessary<br>202 (65%)	Balloon catheter<br>43 (14%)	Non-CR methods: membrane sweep, amniotomy, intravenous oxytocic<br>38 (12%)	Prostaglandin gel/pessary and balloon catheter<br>12 (4%)	Osmotic dilator (eg. Dilapan-S)<br>9 (3%)

For peer review only

Home cervical ripening N=266	Offered option to return home<br><br>39 (15%)	Returned home<br><br>36 (13%)			

Decision making

The questionnaire included a series of questions about choice, decision-making and provision of information when offered IOL (Table 2). Fifty seven percent of women reported that they had either no choice, or no alternative option, about having IOL. While two thirds (66%) felt that options were explained in a way they could understand, only half (50%) felt that they fully understood what to expect during IOL.

Table 2: Choice, decision making and information

**Did you feel you were offered a choice about having your labour induced or waiting for labour to start?**	
Yes, I felt it was fully my decision	122 (39%)
Yes, but I felt there was no other option	117 (38%)
Not really, as I didn't have enough information	10 (3%)
No, I didn't feel I was given a choice	60 (19%)
**Were these options explained to you in a way that you could understand?**	
Yes, I felt I fully understood	205 (66%)
Partly	70 (23%)
Not really	19 (6%)
I'm not sure	2 (0.6%)
No	13 (4%)
**Did you get enough information about what to expect during induction of labour?**	
Yes, I felt I fully understood	155 (50%)
Partly	90 (29%)
Not really	38 (12%)
I'm not sure	0
No	26 (8%)
**Did having an induction lead to any change in your birthplace plans?**	
Yes	148 (48%)
No	153 (59%)
I'm not sure	8 (3%)

The free text responses describe anxiety around risk to their baby's wellbeing as a major influence on the decision to accept the offer of an IOL. For some women this risk was communicated in a way that that contributed to them feeling their choice about IOL was limited.

For peer review only - http://bmjopen.bmj.com/site/about/guidelines.xhtml

“I was induced because of my age. Whilst it was made clear that the decision was my choice, I also felt a lot of pressure from health professionals to be induced” {Participant 010, Multip, Hospital CR}

“It was never something I had a choice in… I was told if I didn’t get induced there was a high chance of my baby being stillborn because I was almost 42 weeks, so this scared me.” {Participant 201, Primip, Home CR}

One hundred and forty-eight (48%) respondents stated that having an IOL changed their plans for labour and birth. Changes included: unable to use water immersion; change of planned place of birth to an obstetric unit from midwifery led unit (MLU) or home birth. Women also reported needing previously unwanted interventions including electronic fetal monitoring and intravenous oxytocin

“I would have liked a water birth but was told it was no longer an option” {Participant 080, Primip, Hospital CR}

Time spent in hospital and at home during cervical ripening

Of the 266 respondents who had CR 39 (15%) were given the option to return home and 36 (14%) did return home. Of those, 22 (61%) had their IOL at a single maternity unit where home CR was offered to all women unless contraindicated.

Some women expressed disappointment at not having the option to return home, whereas others would not have wanted this option. Common themes were lack of choice about where CR occurred and feeling safer in hospital.

“I was told I could have balloon induction and go home at consultant appointment, then when I attended hospital was told this wasn't actually something I could have.” {Participant 100, Primip, Hospital CR}

“I am pleased I didn’t have a choice {of home CR} and stayed overnight. I did have a comfort I was in the right place.” {Participant 081, Primip, Hospital CR}

For women who remained in hospital, the median duration in the antenatal area, between commencing CR and transfer to labour suite, was 22 hours. One hundred women (43%) reported being in antenatal area for 24 hours or longer and 42 (18%) for 48 hours or more. The longest duration of antenatal stay after CR commenced until transfer to a labour room was 260 hours; 11 days. Those who returned home remained at home for a median of 24hours (range: 3 to 168 hours).

The respondents described delays at almost every stage of the IOL process. The most impactful was the wait to be transferred from antenatal area to labour ward after CR, either for artificial rupture of

For peer review only - http://bmjopen.bmj.com/site/about/guidelines.xhtml

the fetal membranes (ARM) or because they were in labour. Staffing was frequently mentioned in relation to delays.

"The staff were pushed to the brink which is why I was in hospital for 11 days before my waters were broken." {Participant 120, Primip, Hospital CR}

For some women the delay between the decision being made for IOL and the process being started, and subsequent delays after IOL commenced, conflicted with the information that their baby was at risk of death if the pregnancy continued and needed to be born soon.

"When you have been told for 3 months that your baby could be in danger if you reach 39 weeks and then have to go beyond that because they don't have a bed for you, it's a very scary time" {Participant 080, Primip, Hospital CR}

Some women described care being planned around service capacity rather than in line with guidance. At times this was described as having a direct effect on their IOL progress.

"Had balloon induction 8am Monday. Balloon out 8am Tuesday and was 2-3cm. However was sent home as there were not enough midwives to induce me further… Was taken back in on Thursday 4pm… 7am Friday taken to the delivery room… at that point was then back to 1cm." {Participant 194, Primip, Home CR}

Women often described poor experience of time spent in antenatal areas during CR: lack of privacy, lack of sleep, lack of food. They also reported a shortfall in support that midwives were able to provide before transfer to labour suite, manifested in lack of appropriate pain relief, lack of emotional support and concerns about clinical care.

"I was labouring behind a curtain, no privacy, others all around me… It was really hard to focus and stay calm and relax with no privacy of my own, no pain relief and no food." {Participant 036, Multip, Hospital CR}

"I spent 3 days crying in pain unable to eat or sleep in hospital" {Participant 135, Primip, Hospital CR}

Among women who stayed in hospital throughout the induction process 196 (74%) had a birth partner who stayed with them compared to 40 (98%) women who returned home. Free text responses indictated that when CR happened in hospital birth partners were not always able to stay as often as women wanted.

For peer review only - http://bmjopen.bmj.com/site/about/guidelines.xhtml

"It was a lonely experience, my husband was not allowed to come in until I was in active labour" {Participant 137, Multip, Hospital CR}

Presence of their birth partner was very important to the respondents, with their absence described as absence of important support. It was also reported that exclusion of birth partners, usually the other parent, denied them full participation in an important life experience.

"the induction also meant my husband actually missed our son being born because I progressed so quickly" {Participant 225, Multip, Hospital CR}

"My partner and I feel like one of the most important experiences of our lives was stolen from us" {Participant 264, Primip, Hospital CR}

Twenty-eight women described being in established labour for a prolonged period and/or approaching second stage while remaining in the antenatal area.

"I was told I couldn't have [epidural analgesia l] until I moved to labour ward but I couldn't move to labour ward as it was full. I was only moved when I was pushing" {Participant 113, Primip, Hospital CR}

Experience of labour induction

The findings suggest that for many women IOL, regardless of any time spent at home, is a period of anxiety, pain and discomfort, and of feeling powerless and lacking control (Table 3). Over a third of women (101) (38% who remained in hospital and 44% of those who went home) did not feel comfortable with their decision to have an IOL, whilst 22% (21% who remained in hospital and 31% who returned home) were worried that IOL might not be safe. Although 36% of those who went home reported anxiety about this, in relation to future choice of home or hospital for CR, more than half in each case (55% hospital and 64% home) said they would choose the same option again.

While 67% of women who stayed in hospital reported having good family support throughout the induction, this was 97% for those who went home.

Table 3: Satisfaction during CR and IOL (Henry et al, 2013)

**From initiation of CR to admission to Labour suite**	**Women who remained in hospital N=227 (3 missing)**		**Women who returned home N=36 (0 missing)**	
	**Agree**	**Unsure & Disagree**	**Agree**	**Unsure & Disagree**
I felt a lot of discomfort	143 (63%)	84 (37%)	28 (78%)	8 (22%)

For peer review only - http://bmjopen.bmj.com/site/about/guidelines.xhtml

I was able to cope with the discomfort	155 (68%)	72 (32%)	29 (81%)	7 (19%)
I felt anxious about being in hospital /going home	115 (51%)	112 (49%)	14 (39%)	22 (61%)
I was able to relax on the AN ward/ at home	101 (44%)	126 (56%)	20 (56%)	16 (44%)
I was able to rest on the AN ward/home	103 (45%)	124 (55%)	24 (67%)	12 (33%)
I had good family support in hospital/home	151 (67%)	76 (33%)	35 (97%)	1 (3%)
I had easy access to information from the staff	127 (56%)	100 (44%)	23 (64%)	13 (36%)
I was worried the induction might not be safe	47 (21%)	180 (79%)	11 (31%)	25 (69%)
I would have preferred to go home/stay at the hospital	97 (43%)	130 (57%)	12 (33%)	24 (67%)
I felt embarrassed by the catheter or gel	21 (9%)	206 (91%)	3 (8%)	33 (92%)
While at home I felt anxious about being at home not hospital	N/A	N/A	13 (36%)	23 (64%)
**IOL**	N= 230		N=36	
I felt anxious about being induced	168 (73%)	62 (26%)	31 (86%)	5 (14%)
I felt in control	62 (27%)	168 (73%)	9 (25%)	27 (75%)
I understood what was happening	174 (76%)	56 (24%)	24 (67%)	12 (33%)
I felt relaxed	62 (27%)	168 (72%)	8 (22%)	28 (78%)
Everything made sense	137 (60%)	93 (40%)	18 (50%)	18 (50%)
I was given clear information	151 (66%)	79 (34%)	17 (47%)	19 (53%)
I felt comfortable with my choice about my care	145 (63%)	85 (37%)	20 (56%)	16 (44%)
I had access to information about the types of induction available	119 (52%)	111 (48%)	20 (56%)	16 (45%)
I had easy access to information about what to do	122 (53%)	108 (47%)	19 (53%)	17 (48%)
I found the induction process uncomfortable	144 (63%)	86 (38%)	32 (89%)	4 (11%)
I was worried about when my labour would begin	176 (76%)	54 (22%)	26 (72%)	10 (28%)

For peer review only

I would choose staying in hospital /going home again	126 (55%)	104 (45%)	23 (64%)	13 (36%)
I would recommend staying in hospital during induction/going home to other women	125 (54%)	105 (36%)	22 (61%)	14 (39%)

Findings related to aspects of participants sense of control are described (Table 4). Overall around one third of women reported feeling like a failure, 40% felt powerless; about half felt fearful and around half felt confident or in control. Most women (both home and hospital) reported feeling that they were with people who cared about them.

Table 4: Sense of Control during induction of labour

	**Agree All respondents N=309**	**Agree In-hospital CR N=230**	**Agree Home CR N=36**
I felt tense	188 (61%)	143 (62%)	23 (64%)
I felt important	215 (69%)	149 (65%)	28 (78%)
I felt confident	149 (48%)	102 (44%)	19 (53%)
I felt in control	140 (45%)	98 (43%)	18 (50%)
I felt fearful	152 (49%)	113 (49%)	19 (53%)
I felt relaxed	95 (31%)	68 (30%)	10 (28%)
I felt good about my behaviour	243 (79%)	183 (80%)	27 (75%)
I felt helpless (powerless)	122 (39%)	92 (40%)	15 (42%)
I felt like a failure	96 (31%)	71 (31%)	12 (33%)
I felt I was with people who care about me	249 (81%)	179 (78%)	31 (86%)

Respondents' feelings of anxiety, powerlessness and lack of control were apparent in free text comments:

"I felt like things happened to me rather than being part of any decisions" {Participant 109, Primip, Hospital CR}

"I felt that choices were taken away from me… I don't think I was given enough information… I wasn't told whether this [painful CR] was normal" {Participant 113, Primip, Hospital CR}

Forty-one women described their experiences of IOL as traumatic and/or having caused significant long-term negative impact on their physical and/or mental wellbeing.

For peer review only - http://bmjopen.bmj.com/site/about/guidelines.xhtml

"It was all so horrendous that I will never have another child. It gives me anxiety thinking about it all. Before this experience I did want more than one child" {Participant 184, Primip, Hospital CR}

Thirty-five women described experiences that were positive overall. Supportive interaction with staff made a significant contribution to women's positive experiences, as did feeling 'safe' and 'cared for'.

"Supportive staff, well informed and felt every decision was genuinely done for our wellbeing" {Participant 092, Primip, Hospital CR}

"My midwife {name} was incredibly supportive throughout labour and birth. We felt safe and cared for." {Participant 040, Primip, Hospital CR}

Discussion

This study reports on the experience of women undergoing CR and IOL more generally, describing wide variation. While some women report a positive experience, significant numbers described a negative experience and a small but important number had an experience that was so traumatic they wished to avoid future, previously planned, pregnancies.

The IOL process begins when a pregnant woman and her care giver first discuss IOL; facilitation of informed decision-making is integral to quality maternity care and prominent within current NICE guidance around IOL (3). However, decision making about IOL may be complex and for many of the women in this survey it seemed poorly supported: most respondents (60%) felt that they either had no choice about IOL or no alternative option. Communicating risk in relation to IOL can be difficult and contentious (12), and this study found that communication around IOL led some women to believe that induction was required to avoid an otherwise high chance of their baby dying. Informed decision making must be underpinned by good quality information, and clinicians should include absolute as well as relative risks of stillbirth when sharing information with women.

Provision of antenatal education and information are recognised as key factors in shaping women's expectations and their ability to cope with labour and birth (13). However, just half (50%) of the survey respondents felt that they fully understood what to expect during their IOL and almost a third (32%) felt unable to cope with the discomfort of CR. Active decision-making may contribute to positive experience when women require previously unwanted interventions (14), however, the women surveyed here described an absence of real choice about IOL alongside significant restrictions on options for care when they accept induction. The most reported restrictions on birth plans were accessing an MLU and use of water during labour or birth. Both are known to improve experience: water is an effective method of pain relief during the first stage of labour (3); births planned in MLUs are associated with significantly reduced intrapartum interventions, with no difference in neonatal outcome (15,16) and increased satisfaction with care (17,18).

Free text responses reveal further impact on experience of care, with women describing a paradox of deciding, or sometimes being persuaded, to have an IOL because of perceived risk but then facing an absence of urgency to commence induction and significant delays during the process itself. This

For peer review only - http://bmjopen.bmj.com/site/about/guidelines.xhtml

was a marked aspect of negative experience and caused stress and anxiety, especially when women reported having been told they required induction because their baby was at risk of stillbirth.

Few women were given the option to return home after CR commenced, limiting our ability to explore whether home CR may improve experience. Of the small number (just 36 women) who did more than half would recommend this option, most were able to cope with the discomfort they felt and most felt able to rest and relax during CR. This is despite most also having had CR using mechanical methods which may be associated with increased initial pain (9,19). Nonetheless, a third of women who went home would have preferred to stay in hospital and 36% felt anxious about being at home rather than in hospital.

Those who experienced CR in hospital describe an environment that frequently failed to meet their needs. Time spent in hospital, during CR and prior to transfer to labour ward, was often characterised by inadequate support from staff and absence of birth partners, combined with insufficient pain relief, lack of privacy in shared wards and failure to take seriously or listen to women's concerns about pain, discomfort and labour progress.

Delays were reported at almost every stage of the IOL process, the most impactful being late assessment of progress and application of further CR agent, and long delays when ready for transfer to labour ward. The women associated delays with poor staffing, reflecting similar recent experience of UK maternity services (20,21). It was not unusual for respondents to feel that their physical safety was compromised; some reporting eventual transfers to delivery suite during advanced labour including second stage. Thus, women who had been informed their baby was at increased risk of death were receiving care below that required once in established labour.

Physical safety and psychological wellbeing are equally valued by women (14) and some respondents reported that they felt the care they received compromised both. Mental ill health is a leading and increasing cause of maternal morbidity and mortality (22). That around half the women (49%) in this survey reported feeling fearful during their IOL, and that many described an experience that was traumatic with lasting negative impact, is of significant concern.

It is of note that there is extensive literature on women's negative experience but much less on the nature of women's positive experience (6). Some women who responded to the survey did have positive experiences of IOL, and the features they describe offer insight to aid understanding of how best to support women undergoing IOL. The most significant factor in women's positive experience was their interaction with staff. This echoes longstanding knowledge of the importance interaction with care givers holds for women's experience (13), and is something that individual practitioners can influence despite organisational and workforce factors.

The overwhelming majority of respondents (81%) stated that they felt that they were with someone who cared about them, but concerningly, nearly 20% of women did not feel this way. Compassionate and respectful care encompasses a sense of care as genuine through 'emotional availability' (6) and it is encouraging that this appears to have been facilitated for most. For many women enabling birth partner attendance during the difficult and often lengthy period of CR would be a simple and effective means of further supporting this.

Limitations

The sampling and recruitment strategies employed meant that it was not possible to determine a denominator from which a response rate could be calculated. It is recognised that this may introduce bias among the characteristics and experiences of those who chose to respond. However, adoption of

For peer review only - http://bmjopen.bmj.com/site/about/guidelines.xhtml

a pragmatic approach to achieve a large sample across multiple NHS sites was deemed to outweigh potential limitations. The results are descriptive and not intended to be generalisable.

The COVID-19 pandemic presented significant challenges, both to study recruitment and to the context in which the respondents received the maternity care they were describing. Maternity unit policies and practices changed in response to various stages of the pandemic, with at least one of the five case study sites halting the offer of home IOL completely. Some placed severe restrictions on the presence of partners on antenatal wards, although it is of note that pre-pandemic it was often usual practice to restrict birth partner attendance to visiting hours only. The survey findings should be understood within this context.

A significant limitation was that so few women were offered the option to return home during CR (n=36, 12%), limiting the opportunity to compare their experience with those who remained in hospital. However, there is very little information about women's experience of home CR and this work adds important and relevant understanding about women's experience of undergoing IOL, cervical ripening in particular, both in hospital and at home.

Conclusion

This work demonstrates that women undergoing IOL do not experience it as a benign, consequence free intervention; induction of labour often causes anxiety and removes options for birth that women had hoped would enhance their experience and outcomes. Assessing risks and benefits when offering, and considering the offer of, an IOL can be complex; clinicians must ensure that women are informed of and understand the absolute as well as relative risks of continuing the pregnancy, along with the risks and consequences of IOL itself.

Experience of childbirth is important to women; known to impact physical, social and psychological short and long-term outcomes. Although some women had a positive experience of IOL, many experienced poor care, inadequate communication and delays during IOL that had the potential to jeopardise their safety or that of their baby. For some, this led to long term psychological maternal morbidity including the desire to avoid future pregnancies.

Returning home during CR may be an option to improve women's experience of IOL; however few women were offered this opportunity and numbers were too small to draw definite conclusions.

Induction of labour rates have been increased with good intentions of reducing rates of stillbirth and severe neonatal morbidity, but this does not always appear to have been accompanied by adequate planning or increase in the facilities and resources that are necessary to provide an effective service. It is crucial that the expected principles of person-centred individualised care, provided with dignity and respect, apply equally to women experiencing IOL. Listening to women and service users, ensuring that practice is based on their needs, and that services are sufficiently resourced are all essential to the provision of safe and effective induction pathways.

Author Contribution Statement

For peer review only - http://bmjopen.bmj.com/site/about/guidelines.xhtml

For peer review only

Dr Mairi Harkness: Substantial contribution to the design of the work, and acquisition, analysis, and interpretation of data for the work. Drafted the work and revised it critically for important intellectual content. Final approval of the version to be published. Agrees to be accountable for all aspects of the work in ensuring that questions related to the accuracy or integrity of any part of the work are appropriately investigated and resolved.

Dr Cassandra Yuill: Substantial contribution to the design of the work, and acquisition, analysis, and interpretation of data for the work. Revised the work critically for important intellectual content. Final approval of the version to be published. Agrees to be accountable for all aspects of the work in ensuring that questions related to the accuracy or integrity of any part of the work are appropriately investigated and resolved.

Professor Helen Cheyne: Substantial contribution to the design of the work, and acquisition, analysis, and interpretation of data for the work. Revised the work critically for important intellectual content. Final approval of the version to be published. Agrees to be accountable for all aspects of the work in ensuring that questions related to the accuracy or integrity of any part of the work are appropriately investigated and resolved.

Professor Christine McCourt: Substantial contribution to the design of the work, and acquisition, analysis, and interpretation of data for the work. Revised the work critically for important intellectual content. Final approval of the version to be published. Agrees to be accountable for all aspects of the work in ensuring that questions related to the accuracy or integrity of any part of the work are appropriately investigated and resolved.

Dr Mairead Black: Substantial contribution to the conception and design of the work, and interpretation of data for the work. Revised the work critically for important intellectual content. Final approval of the version to be published. Agrees to be accountable for all aspects of the work in ensuring that questions related to the accuracy or integrity of any part of the work are appropriately investigated and resolved.

Professor Dharmintra Pasupathy: Substantial contribution to the conception and design of the work. Revised the work critically for important intellectual content. Final approval of the version to be published. Agrees to be accountable for all aspects of the work in ensuring that questions related to the accuracy or integrity of any part of the work are appropriately investigated and resolved.

Professor Julia Sanders: Substantial contribution to the conception and design of the work, and interpretation of the data for the work. Revised the work critically for important intellectual content. Final approval of the version to be published. Agrees to be accountable for all aspects of the work in ensuring that questions related to the accuracy or integrity of any part of the work are appropriately investigated and resolved.

Chloe Wallace: Substantial contribution to the design of the work. Revised the work critically for important intellectual content. Final approval of the version to be published. Agrees to be accountable for all aspects of the work in ensuring that questions related to the accuracy or integrity of any part of the work are appropriately investigated and resolved.

Neelam Heera-Shergill: Substantial contribution to the design of the work. Revised the work critically for important intellectual content. Final approval of the version to be published. Agrees to be accountable for all aspects of the work in ensuring that questions related to the accuracy or integrity of any part of the work are appropriately investigated and resolved.

For peer review only - http://bmjopen.bmj.com/site/about/guidelines.xhtml

Professor Sarah Stock: Substantial contribution to the conception and design of the work, and interpretation of data for the work. Revised the work critically for important intellectual content. Final approval of the version to be published. Agrees to be accountable for all aspects of the work in ensuring that questions related to the accuracy or integrity of any part of the work are appropriately investigated and resolved.

Competing Interests Statement

The authors declare the following competing interests

Support for the present manuscript: The CHOICE Study was funded by National Institute of Healthcare Research Health Technology and Assessment (NIHR HTA). The grant was paid to institution and the authors have the following associations: Patient and public involvement (PPI) Group member (paid); Funded research fellows (2); Principal investigator and co-investigators with funded time.

CHOICE study budget has been used by some authors for travel and conference cost.

Authors hold additional, paid to institution, grants from: NIHR; Wellcome Trust; Medical Research Council; Chief Scientist Office of Scotland; Tommy's Charity; Scottish government; Aberlour Childcare Trust (small project grant); University of Stirling article processing fund.

Authors declare the following: Consultancy fees: Natera, consultancy on pre-term birth treatments (paid to institution); Honoria: Hologic, Honoria for educational talk (paid to institution); Expert testimony: Expert witness in (midwifery) in civil litigation claims (self employed)

Authors declare the following participation on data safety monitoring or advisory boards: Membership of several NIHR Trial Steering committees; NIHR Health Technology and Assessment DMC and TSC

Authors are unpaid members or trustees of the following Boards and Committees: National Institute of Health and Care Excellence Antenatal Guideline Group 2018-202; Cysters (CIO) Chair of trustees; Platform Housing Group, trainee Board role; UK Government Period Poverty Task Force, member; UK sepsis Trust, Trustee

Funding Statement

The CHOICE Study was funded by the National Institute of Healthcare Research Health Technology and Assessment (NIHR HTA) NIHR127569. The views expressed are those of the authors and not necessarily those of the NIHR or the Department of Health and Social Care.

Data sharing statement

The data sets generated during this study are available from the corresponding author upon reasonable request.

For peer review only - http://bmjopen.bmj.com/site/about/guidelines.xhtml

Ethics Approval

Ethics approval was obtained from the York & Humber – Sheffield Research Ethics Committee in June 2020 (IRAS: 276788) as part of the CHOICE Study application.

Acknowledgments

Grateful thanks are due to the women who completed the survey and shared their experiences and the NHS staff who supported the CHOICE Study.
The authors would also like to thank the CHOICE Study Team.

References

1. NMPA Project Team. *National Maternity and Perinatal Audit: Clinical Report 2022. Based on births in NHS maternity services in England and Wales between 1 April 2018 and 31 March 2019.* London: RCOG; 2022.
2. Harkness, M., Yuill, C., Cheyne, H, Stock, S, J, McCourt, C. On behalf of the CHOICE Study Consortia. *Induction of labour during the COVID-19 pandemic: a national survey of impact on practice in the UK.* BMC Pregnancy Childbirth 21, 310 (2021). https://doi.org/10.1186/s12884-021-03781-x
3. National Institute for Clinical Excellence NICE. *Inducing Labour. Clinical Guideline {CG207}.* Published 4th November 2021. At: https://www.nice.org.uk/guidance/ng207
4. Coates, R, Cupples, G, Scamell, A, McCourt, C, Bhide, A. *Women's experiences of outpatient induction of labour with double balloon catheter or prostaglandin pessary: A qualitative study.* Women and Birth. 2021. 34(4):e406-e415. doi: 10.1016/j.wombi.2020.07.006.
5. WHO recommendations: intrapartum care for a positive childbirth experience. Geneva: World Health Organization; 2018. Licence: CC BY-NC-SA 3.0 IGO.
6. Vedeler C, Nilsen ABV, Blix E, Downe S, Eri TS. What women emphasise as important aspects of care in childbirth–an online survey. BJOG 2022: 128; 647-655
7. Bell A,F, Andersson, E. *The birth experience and women's postnatal depression: a systematic review.* Midwifery. 2016. 39:112-123
8. Stock SJ, Bhide A, Richardson H, et al. *Cervical ripening at home or in hospital—prospective cohort study and process evaluation (CHOICE) study: a protocol.* BMJ Open 2021;11:e050452. doi:10.1136/bmjopen-2021-050452
9. Henry, A., Madan, A., Reid, R. et al. *Outpatient Foley catheter versus inpatient prostaglandin E2 gel for induction of labour: a randomised trial.* BMC Pregnancy Childbirth. 2013. 13, 25. https://doi.org/10.1186/1471-2393-13-25

For peer review only - http://bmjopen.bmj.com/site/about/guidelines.xhtml

10. Hodnett ED, Simmons-Tropea DA. *The Labour Agentry Scale: psychometric properties of an instrument measuring control during childbirth*. Res Nurs Health. 1987;10(5):301-10.
11. Scottish Government. *Maternity care survey 2018: national results.* Scottish Government Publications. 2019. ISBN: 9781787816398. At: www.gov.scot/publications/maternity-care-survey-2018-national-results/pages/11/
12. Cheyne, H, Abhyankar P, Williams B. *Elective induction of labour: The problem of interpretation and communication of risks.* Midwifery. 2012 http://dx.doi.org/10.1016/j.midw.2012.06.009
13. Borrelli SE, Walsh D, Spiby H. *First-time mothers' expectations of the unknown territory of childbirth: Uncertainties, coping strategies and 'going with the flow*. Midwifery. 2018. 63:39-45
14. Downe S, Finlayson K, Oladapo OT, Bonet M, Gülmezoglu AM. *Correction: What matters to women during childbirth: A systematic qualitative review.* PLOS ONE. 2018. 13(5): e0197791. https://doi.org/10.1371/journal.pone.0197791
15. Birthplace. *Perinatal and maternal outcomes by planned place of birth for healthy women with low risk pregnancies: the Birthplace in England national prospective cohort study.* BMJ 2011; 343:d7400. doi: https://doi.org/10.1136/bmj.d7400 (Published 25 November 2011)
16. Scarf VL, Rossiter C, Vedam S, Dahlen HG, Ellwood D, Forster D, Foureur MJ, McLachlan H, Oats J, Sibbritt D, Thornton C, Homer CSE. *Maternal and perinatal outcomes by planned place of birth among women with low-risk pregnancies in high-income countries: A systematic review and meta-analysis.* Midwifery. 2018 Jul;62:240-255. doi: 10.1016/j.midw.2018.03.024. Epub 2018 Apr 3. PMID: 29727829.
17. Overgaard, C., Fenger-Grøn, M., & Sandall, J. *Freestanding midwifery units versus obstetric units: Does the effect of place of birth differ with level of social disadvantage?* BMC Public Health. 2012. 12(1). https://doi.org/10.1186/1471-2458-12-478
18. Macfarlane, A.J., Rocca-Ihenacho, L., Turner, L. and Roth, C. *Survey of women's experiences of care in a new freestanding midwifery unit in an inner city area of London, England – 1: Methods and women's overall ratings of care.* Midwifery. 2014. 30(9), pp.998-1008.
19. Coates, R, Cupples, G, Foya V, McCourt C, Scamell M. *Women's experiences of induction of labour: qualitative systematic review and thematic synthesis.* Midwifery, 2018-10. DOI: 10.1016/j.midw.2018.10.013
20. RCM. *RCM warns of midwife exodus as maternity staffing crisis grows.* Media Release. RCM October 2021. At: https://www.rcm.org.uk/media-releases/2021/september/rcm-warns-of-midwife-exodus-as-maternity-staffing-crisis-grows/
21. Gupta JK, Maher A, Stubbs C, Brocklehurst P, Daniels JP, Hardy P. *Synthetic Osmotic Cervical Dilator for Induction of Labor in Comparison to Dinoprostone Vaginal insErt (SOLVE) collaborative group. A randomized trial of synthetic osmotic cervical dilator for induction of labor vs dinoprostone vaginal insert.* Am J Obstet Gynecol MFM. 2022 Jul;4(4):100628. doi: 10.1016/j.ajogmf.2022.100628. Epub 2022 Mar 28. Erratum in: Am J Obstet Gynecol MFM. 2022 Aug 9;:100702. PMID: 35358740.
22. Knight M, Bunch K, Patel R, Shakespeare J, Kotnis R, Kenyon S, Kurinczuk JJ (Eds.). *Saving Lives, Improving Mothers' Care. Core report: Lessons learned to inform maternity care from the UK and Ireland Confidential Enquiries into Maternal Deaths and Morbidity 2018-20.* November 2022

For peer review only - http://bmjopen.bmj.com/site/about/guidelines.xhtml

For peer review only

For peer review only - http://bmjopen.bmj.com/site/about/guidelines.xhtml

Cervical Ripening at Home or In-Hospital (qCHOICE) Study Induction of Labour Experience Questionnaire

You are being invited to participate in a research study titled qCHOICE. This study is being done by University of Edinburgh, Professor Helen Cheyne from University of Stirling and Professor Christine McCourt from City, University of London.

Many pregnant women will be offered Induction of Labour (IOL) towards the end of their pregnancy. Labour often starts on its own, but sometimes it needs to be started artificially, usually because the baby is late. The first part of this process is called 'cervical ripening' (sometimes also called cervical priming), where medication or a specialised balloon is used to soften and open the cervix (neck of the womb), getting it ready for labour.

Cervical ripening used to be performed only in hospitals. However, about half of maternity units in the UK now offer 'home cervical ripening' – where women have the procedure started off in hospital, but can spend some time at home whilst waiting for the treatment to work.

We want to ask women about their experiences of cervical ripening (at home or in hospital) and having their labour induced. You are being invited to take part in the qCHOICE study because you had induction of labour.

This survey asks about you and your experiences of induction of labour and your labour and birth. What you tell us is very important; it helps us find out whether going home or staying in hospital for the first part of induction (cervical ripening) is acceptable to women, how good your care was and how it might be improved. **The survey will take you approximately 20 mintes to complete.**

Further details about the study, including information about data protection, are available in the participant information sheet that you can read and download here:

Participant Information Sheet (PIS)

Data Protection Information Sheet

For peer review only - http://bmjopen.bmj.com/site/about/guidelines.xhtml

Please read the participant information sheet and keep a copy before starting the survey. If you have any questions, you can contact either Dr Cassandra Yuill or Dr Mairi Harkness.

If you would prefer us to post you a paper copy to complete, or if you would prefer a researcher to complete it with you over the telephone, or need an interpreter to do this, please e-mail or call us:

Dr Cassandra Yuill	Dr Mairi Harkness
E: cassandra.yuill@city.ac.uk	E: mairi.harkness@stir.ac.uk
T: 07840872417	T: 01786466119

There are no right or wrong answers, we just would like to find out your views and feelings and welcome your honesty. All your answers are treated as private.

For peer review only

For peer review only - http://bmjopen.bmj.com/site/about/guidelines.xhtml

Have you had an induction of labour and given birth to your baby yet? ✱ *Required*

- ○ Yes
- ○ No

For peer review only

For peer review only - http://bmjopen.bmj.com/site/about/guidelines.xhtml

Thank you for your interest in taking part in the Cervical Ripening at Home or In-Hospital Process Evaluation (qCHOICE). You have been redirected because you have not yet had your baby.

If you do end up having an induction of labour, you will receive a notification from your online Maternity Notes about this questionnaire 10 days after you have given birth. The notification will provide a link back to this site.

For peer review only

CONSENT FORM for Service Users

If you are happy to take part by completing this survey, please *initial* the boxes below to confirm your consent to participate:

	Please initial each box ✱ *Required*
1. I confirm that I have read and understand the information sheet (05 MAY 2020 Version 2.0) and the Data Protection Information Sheet (01 APR 2020 Version 1.0) for the above study. I have had the opportunity to consider the information, ask questions and have had these questions answered satisfactorily.	
2. I understand that my participation is voluntary and that I am free to withdraw at any time, without giving any reason and without my medical care and/or legal rights being affected.	
3. I give permission for my personal information (including name, address, date of birth, telephone number and consent form) to be passed to the University of Stirling and City, University of London for administration of the study.	
4. I agree to take part in the above study.	

Name of Person Giving Consent ✱ *Required*

Date ✱ *Required*

Dates need to be in the format 'DD/MM/YYYY', for example 27/03/1980.

For peer review only - http://bmjopen.bmj.com/site/about/guidelines.xhtml

(dd/mm/yyyy)

For peer review only - http://bmjopen.bmj.com/site/about/guidelines.xhtml

First some brief questions about you and the care you received. It is important to fill these out as you will then be asked questions relevant to the type of care you had.

How many weeks pregnant were you when you were induced? ✱ *Required*

What was your baby's birthweight in grams? *Example: 3400*

Please enter a number.

Is this your first baby? ✱ *Required*

- Yes
- No

What hospital did you give birth in? ✱ *Required*

What is your age? ✱ *Required*

For peer review only - http://bmjopen.bmj.com/site/about/guidelines.xhtml

What is your postcode? *Example: A12 3BC* ✱ *Required*

Please enter a valid UK postcode.

What is your ethnic group?

- White
- Mixed/Multiple ethnic groups
- Asian/Asian British
- Black/African/Caribbean/Black British
- Other ethnic group

White

- English/Welsh/Scottish/Northern Irish/British
- Irish
- Gypsy or Irish Traveller
- Any other White background

Mixed/Multiple ethnic groups

- White and Black Caribbean
- White and Black African
- White and Asian
- Any other Mixed/Multiple ethnic background

Asian/Asian British

- Indian
- Pakistani
- Bangladeshi
- Chinese
- Any other Asian background

Black/African/Caribbean/Black British

- African
- Caribbean
- Any other Black/African/Caribbean background

Other ethnic group

- Arab
- Other

If you selected Other, please specify:

For peer review only - http://bmjopen.bmj.com/site/about/guidelines.xhtml

The following questions ask about your choices about induction of labour

What was the main reason your obstetrician or midwife recommended induction of labour? ✱ *Required*

- ○ Length of pregnancy
- ○ Medical reasons (e.g. high blood pressure)
- ○ I'm not sure/don't know
- ○ Other

If you selected Other, please specify:

Before the decision for induction, where had you planned or expected to have your baby?

- ○ In a hospital delivery suite (labour ward with obstetricians)
- ○ In a hospital-based midwife-led unit/birth centre (maternity unit run by midwives only)
- ○ In a 'freestanding' midwife-led unit/birth centre (maternity unit outside of hospital run by midwives only)
- ○ At home
- ○ I hadn't decided yet

For peer review only - http://bmjopen.bmj.com/site/about/guidelines.xhtml

Did having an induction lead to any change in your birthplace plans?

- Yes
- No
- I'm not sure

If yes, please explain the reason.

Did you feel you were offered a choice about having your labour induced or waiting for labour to start?

- Yes, I felt this was fully my decision
- Yes, but I felt there was no other option
- Not really, as I didn’t feel I had enough information
- No, I didn't feel I was given a choice

Were your options explained to you in a way that you could understand?

- Yes, I felt I fully understood the options and their risks or benefits
- Partly
- Not really
- I’m not sure
- No

For peer review only - http://bmjopen.bmj.com/site/about/guidelines.xhtml

Thinking about what happened when you had your labour induced

Did you get enough information about what to expect during induction of labour?

- Yes, I felt I fully understood the what to expect
- Partly
- Not really
- I'm not sure
- No

What method was used to start your labour? This may have also been called cervical ripening or priming. *Tick all that apply.* ✱ *Required*

- Catheter (or balloon)
- Gel pessaries (e.g. Propess or Prostin)
- I'm not sure/don't know
- Other

If you selected Other, please specify:

Thinking about *when the catheter or gel was first inserted* how much do you agree or disagree with the following:

	Strongly agree	Agree	Unsure	Disagree	Strongly disagree

For peer review only - http://bmjopen.bmj.com/site/about/guidelines.xhtml

I felt a lot of discomfort	☐	☐	☐	☐	☐
I was able to cope with the discomfort	☐	☐	☐	☐	☐
I felt tense and anxious during the insertion	☐	☐	☐	☐	☐
I felt anxious that the induction wouldn't work	☐	☐	☐	☐	☐

Henry, A., Madan, A., Reid, R. et al. Outpatient Foley catheter versus inpatient prostaglandin E2 gel for induction of labour: a randomised trial. BMC Pregnancy and Childbirth 13, 25 (2013).

After the catheter or gel was inserted to start the process, did you have monitoring of your baby's heart (with a belt, CTG or doppler)?

- ○ Yes
- ○ No

How long was this for (minutes)? *Don't worry about exact times but recall as best you can.*

Please enter a whole number (integer).

____

Were you offered the choice to go home for the first part of the process (cervical ripening or priming)? ✱ *Required*

- ○ Yes
- ○ No

For peer review only - http://bmjopen.bmj.com/site/about/guidelines.xhtml

If yes, did you go home? ✱ *Required*

- Yes
- No
- I was not offered the option to go home

If you didn’t go home, what was the main reason?

- I didn’t want to go home
- I initially wanted to go home but I changed my mind
- I was recommended to stay after the initial monitoring

For peer review only - http://bmjopen.bmj.com/site/about/guidelines.xhtml

For women who remained in hospital

If you stayed in hospital how long did you stay in antenatal unit before admission to labour ward or birth centre? *Don't worry about exact times but recall as best you can.*

Hours		Please select
Minutes		Please select

Did anyone (e.g. birth partner) stay with you during the time before your admission to the labour ward or birth centre?

- No
- Birth partner
- Other

If you selected Other, please specify:

If you have other children, did you use paid or unpaid childcare (other than their primary carers) during the time you stayed in the hospital? *Childcare includes private or public nursery, a paid or unpaid relative, friend or babysitter.*

- Yes
- No

If yes, how many hours of childcare were required while you were in hospital?

For peer review only - http://bmjopen.bmj.com/site/about/guidelines.xhtml

- [ ] Paid
- [ ] Unpaid

Estimated additional hours? (paid)

Please enter a whole number (integer).

Estimated additional hours? (unpaid)

Please enter a whole number (integer).

What was your mode of transport to and from the hospital?

- [ ] Ambulance
- [ ] Public transportation
- [ ] Car
- [ ] Taxi
- [ ] Other

If you selected Other, please specify:

Please estimate how much you spent travelling to and from the hospital. If you are unsure, please provide an estimation on petrol/diesel used, parking expenses or bus fare.

For peer review only - http://bmjopen.bmj.com/site/about/guidelines.xhtml

£

What is your birth partner's employment status?

- Not in paid employment (e.g. looking after children/home; unemployed)
- In paid full-time employment
- In paid part-time employment
- Self-employed
- Other

If you selected Other, please specify:

Thinking about *the time from when the first dose of gel or catheter was inserted on the antenatal ward to the time you went to labour ward or birth centre*, how much do you agree or disagree with the following:

	Strongly agree	Agree	Unsure	Disagree	Strongly disagree
I felt a lot of discomfort	☐	☐	☐	☐	☐
I was able to cope with the discomfort	☐	☐	☐	☐	☐
I felt anxious about being in hospital	☐	☐	☐	☐	☐
I was able to relax on the antenatal ward	☐	☐	☐	☐	☐

For peer review only - http://bmjopen.bmj.com/site/about/guidelines.xhtml

I was able to rest on the antenatal ward	☐	☐	☐	☐	☐
I had good family support in hospital	☐	☐	☐	☐	☐
I had easy access to information from the staff	☐	☐	☐	☐	☐
I was worried the induction might not be safe	☐	☐	☐	☐	☐
I would have preferred to have gone home	☐	☐	☐	☐	☐
I felt embarrassed by the catheter/gel	☐	☐	☐	☐	☐

Henry, A., Madan, A., Reid, R. et al. Outpatient Foley catheter versus inpatient prostaglandin E2 gel for induction of labour: a randomised trial. BMC Pregnancy and Childbirth 13, 25 (2013).

Thinking about how you felt about your induction of labour overall

For each of the following statements, please tick the option which shows how you felt about your induction.

	Strongly agree	Agree	Unsure	Disagree	Strongly disagree
I felt anxious about being induced	☐	☐	☐	☐	☐
I felt in control	☐	☐	☐	☐	☐
I understood what was happening	☐	☐	☐	☐	☐
I felt relaxed	☐	☐	☐	☐	☐

For peer review only - http://bmjopen.bmj.com/site/about/guidelines.xhtml

Everything made sense	☐	☐	☐	☐	☐
I was given clear information	☐	☐	☐	☐	☐
I felt comfortable with my choice about my care	☐	☐	☐	☐	☐
I had access to information about the types of induction available	☐	☐	☐	☐	☐
I had easy access to information about what to do	☐	☐	☐	☐	☐
I found the induction process uncomfortable	☐	☐	☐	☐	☐
I was worried about when my labour would begin	☐	☐	☐	☐	☐
I would choose staying in hospital again	☐	☐	☐	☐	☐
I would recommend staying in hospital during induction to other women	☐	☐	☐	☐	☐

Henry, A., Madan, A., Reid, R. et al. Outpatient Foley catheter versus inpatient prostaglandin E2 gel for induction of labour: a randomised trial. BMC Pregnancy and Childbirth 13, 25 (2013).

For women who went home

After the catheter or gel was inserted and you went home, how long did you stay at home before admission to labour ward or birth centre? *Don't worry about exact times but recall as best you can.*

Hours		Please select
Minutes		Please select

Did you phone the hospital ward or your midwife for advice while at home?

- Yes
- No

If yes, how many times?

Please enter a whole number (integer).

Did you return to the hospital but go home again without being admitted to the labour ward or birth centre?

- Yes
- No

If yes, how many times?

Please enter a whole number (integer).

For peer review only - http://bmjopen.bmj.com/site/about/guidelines.xhtml

Did anyone (e.g. birth partner) stay with you during your time at home before your admission to the labour ward or birth centre?

- ○ No
- ○ Birth partner
- ○ Other

If you selected Other, please specify:

If you have other children, did you use paid or unpaid childcare (other than primary carers) during the time you stayed at home after cervical ripening/priming?
Childcare includes private or public nursery, a paid or unpaid relative, friend or babysitter.

- ○ Yes
- ○ No

If yes, how many hours of childcare were required while you were at home prior to admission to the labour ward?

- ☐ Paid
- ☐ Unpaid

Estimated additional hours? *(paid)*

For peer review only - http://bmjopen.bmj.com/site/about/guidelines.xhtml

Please enter a whole number (integer).

Estimated additional hours? *(unpaid)*

Please enter a whole number (integer).

What was your mode of transport to and from the hospital?

- ☐ Ambulance
- ☐ Public transportation
- ☐ Car
- ☐ Taxi
- ☐ Other

If you selected Other, please specify:

Please estimate how much you spent travelling to and from the hospital. If you are unsure, please provide an estimation on petrol/diesel used, parking expenses or bus fare.

£

What is your birth partner's employment status?

For peer review only - http://bmjopen.bmj.com/site/about/guidelines.xhtml

- Not in paid employment (e.g. looking after children/home; unemployed)
- In paid full-time employment
- In paid part-time employment
- Self-employed
- Other

If you selected Other, please specify:

Thinking about *the time from when you went home until the time you came back in to hospital*, how much do you agree or disagree with the following:

	Strongly agree	Agree	Unsure	Disagree	Strongly disagree
I felt a lot of discomfort	☐	☐	☐	☐	☐
I was able to cope with the discomfort	☐	☐	☐	☐	☐
I felt anxious about going home	☐	☐	☐	☐	☐
While at home I felt anxious being at home rather than in hospital	☐	☐	☐	☐	☐
I was able to relax at home	☐	☐	☐	☐	☐
I was able to rest at home	☐	☐	☐	☐	☐
I had good family support at home	☐	☐	☐	☐	☐

For peer review only - http://bmjopen.bmj.com/site/about/guidelines.xhtml

I had easy access to information from the hospital	☐	☐	☐	☐	☐
I was worried it might not be safe to be at home	☐	☐	☐	☐	☐
I would have preferred to stay at the hospital	☐	☐	☐	☐	☐
I felt embarrassed by the catheter/gel	☐	☐	☐	☐	☐

Henry, A., Madan, A., Reid, R. et al. Outpatient Foley catheter versus inpatient prostaglandin E2 gel for induction of labour: a randomised trial. BMC Pregnancy and Childbirth 13, 25 (2013).

Thinking about how you felt about your induction of labour overall

For each of the following statements, please tick the option which shows how you felt about your induction.

	Strongly agree	Agree	Unsure	Disagree	Strongly disagree
I felt anxious about being induced	☐	☐	☐	☐	☐
I felt in control	☐	☐	☐	☐	☐
I understood what was happening	☐	☐	☐	☐	☐
I felt relaxed	☐	☐	☐	☐	☐
Everything made sense	☐	☐	☐	☐	☐
I was given clear information	☐	☐	☐	☐	☐

For peer review only - http://bmjopen.bmj.com/site/about/guidelines.xhtml

I felt comfortable with my choice about my care	☐	☐	☐	☐	☐
I had access to information about the types of induction available	☐	☐	☐	☐	☐
I had easy access to information about what to do	☐	☐	☐	☐	☐
I found the induction process uncomfortable	☐	☐	☐	☐	☐
I was worried about when my labour would begin	☐	☐	☐	☐	☐
I would choose going home again	☐	☐	☐	☐	☐
I would recommend going home during induction to other women	☐	☐	☐	☐	☐

Henry, A., Madan, A., Reid, R. et al. Outpatient Foley catheter versus inpatient prostaglandin E2 gel for induction of labour: a randomised trial. BMC Pregnancy and Childbirth 13, 25 (2013).

For peer review only - http://bmjopen.bmj.com/site/about/guidelines.xhtml

Thinking about your time in the labour ward or birth centre

Thinking now about the induction of labour from *the time you were admitted to the labour ward or birth centre to the time the baby was born*, how much do you agree or disagree with the following:

	Strongly agree	Agree	Unsure	Disagree	Strongly disagree
I felt a lot of discomfort	☐	☐	☐	☐	☐
I was able to cope with the discomfort	☐	☐	☐	☐	☐
I felt tense and anxious	☐	☐	☐	☐	☐
I felt anxious that the induction wouldn't work	☐	☐	☐	☐	☐
I felt that my labour had started	☐	☐	☐	☐	☐

Henry, A., Madan, A., Reid, R. et al. Outpatient Foley catheter versus inpatient prostaglandin E2 gel for induction of labour: a randomised trial. BMC Pregnancy and Childbirth 13, 25 (2013).

Your feelings about your labour and birth overall. Please try to rate each statement on its own. Do not consider the other statements. Mark the position of the statement which relates most closely to your childbirth experience.

	Almost all of the time	A lot but not always	A little more than half the time	About half the time	Slightly less than half the time	Some-times Never or almost never
I felt tense	☐	☐	☐	☐	☐	☐

For peer review only - http://bmjopen.bmj.com/site/about/guidelines.xhtml

I felt important	☐	☐	☐	☐	☐	☐
I felt confident	☐	☐	☐	☐	☐	☐
I felt in control	☐	☐	☐	☐	☐	☐
I felt fearful	☐	☐	☐	☐	☐	☐
I felt relaxed	☐	☐	☐	☐	☐	☐
I felt good about my behaviour	☐	☐	☐	☐	☐	☐
I felt helpless (powerless)	☐	☐	☐	☐	☐	☐
I felt like a failure	☐	☐	☐	☐	☐	☐
I felt I was with people who care about me	☐	☐	☐	☐	☐	☐

The 'Labour Agentry Scale' (LAS). Hodnett & Simmons Tropea, 1987.

How many nights did you stay in the hospital or birth centre after your baby was born?

After going home following the birth of your baby, did you return to the hospital and stay over night for reasons related to your baby or the birth?

- ◯ Yes
- ◯ No

If yes, how many nights?

For peer review only - http://bmjopen.bmj.com/site/about/guidelines.xhtml

How many weeks old is your baby TODAY?

For peer review only - http://bmjopen.bmj.com/site/about/guidelines.xhtml

Your health and wellbeing since the birth of your baby

Below are some statements about feelings and thoughts. Please tick the box that best describes your experience of each over the last 2 weeks.

	None of the time	Rarely	Some of the time	Often	All of the time
I've been feeling optimistic about the future	☐	☐	☐	☐	☐
I've been feeling useful	☐	☐	☐	☐	☐
I've been feeling relaxed	☐	☐	☐	☐	☐
I've been dealing with problems well	☐	☐	☐	☐	☐
I've been thinking clearly	☐	☐	☐	☐	☐
I've been feeling close to other people	☐	☐	☐	☐	☐
I've been able to make up my own mind about things	☐	☐	☐	☐	☐

The Short Warwick-Edinburgh Mental Well-being Scale (SWEMWBS) © NHS Health Scotland, University of Warwick and University of Edinburgh, 2007, all rights reserved.

There have been many changes in maternity services due to COVID-19. Did your feelings about induction of labour change at all due the COVID-19 pandemic?

○ Yes

○ No

If yes, please provide details:

If you were offered at home cervical ripening, did your feelings about the choice to go home/stay in hospital after catheter or gel insertion change at all due to COVID-19 pandemic?

○ Yes
○ No
○ Not applicable

If yes, please provide details:

Please add any other comments about how your experience of induction or feelings about it were affected, if at all, by the COVID-19 pandemic.

For peer review only - http://bmjopen.bmj.com/site/about/guidelines.xhtml

Thinking back about your induction and birth experiences, is there anything else you would like to tell us?

For peer review only

For peer review only - http://bmjopen.bmj.com/site/about/guidelines.xhtml

The CHOICE study collects de-identified information about the care of all women who had induction of labour in hospitals taking part. We would like your permission to add your survey results to this information. Your information will be used only for research and will not identify you individually.

If you give your permission to add your survey results to this information it will not be shared with the people who looked after you and will in no way affect your current or future treatment or care.

Do you give your permission for CHOICE study researchers to add your survey results to information held about your hospital stay? ✱ *Required*

- Yes
- No

Before you finish

We also hope to speak with a small number of women (and their birth partners) about their experiences of induction of labour and birth.

Completing this survey does not mean you have to take part in an interview. If you are happy for one of the research team to contact you to talk about a possible interview, please enter your contact details below. You do not have to make up your mind about this now. This just gives us permission to call you with more information.

I'm happy for a research team member to call me to about a possible interview. ✱ *Required*

- Yes
- No

My phone number

For peer review only - http://bmjopen.bmj.com/site/about/guidelines.xhtml

Please enter a valid phone number.

My email address (if preferred)

I would like you to send me a summary of the final project report. ✱ *Required*

- Yes
- No

Your email address

Women and partners who take part in our interviews will receive a £10 voucher as a thanks for giving the additional time and support to our study.

For peer review only - http://bmjopen.bmj.com/site/about/guidelines.xhtml

Your responses have been submitted.

Thank you for taking part in the qCHOICE Study. We appreciate your participation.

If you have shown interest in taking part in an interview, a member of the qCHOICE Study team will get in touch with you soon to follow-up about this.

For peer review only

Cervical Ripening at Home or In-Hospital Process Evaluation (qCHOICE) Contact

If you have any questions, please email or call us:

Dr Cassandra Yuill
E: cassandra.yuill@city.ac.uk
T: 07840872417

Dr Mairi Harkness
E: mairi.harkness@stir.ac.uk
T: 01786466119

Key for selection options

5 - How many weeks pregnant were you when you were induced?

37
38
39
40
41
42
43

9 - What is your age?

16
17
18
19
20
21
22
23
24
25
26
27
28
29
30

For peer review only - http://bmjopen.bmj.com/site/about/guidelines.xhtml

31
32
33
34
35
36
37
38
39
40
41
42
43
44
45
46
47
48
49
50
51
52
53
54
55
56
57
58
59

23.1.b -

0
1
2
3
4
5
6
7
8

For peer review only - http://bmjopen.bmj.com/site/about/guidelines.xhtml

For peer review only

For peer review only - http://bmjopen.bmj.com/site/about/guidelines.xhtml

23.2.b -

For peer review only - http://bmjopen.bmj.com/site/about/guidelines.xhtml

27
28
29
30
31
32
33
34
35
36
37
38
39
40
41
42
43
44
45
46
47
48
49
50
51
52
53
54
55
56
57
58
59

31.1.b -

0
1
2
3

For peer review only

For peer review only - http://bmjopen.bmj.com/site/about/guidelines.xhtml

For peer review only

For peer review only - http://bmjopen.bmj.com/site/about/guidelines.xhtml

44
45
46
47
48
49
50
51
52
53
54
55
56
57
58
59

31.2.b -

0
1
2
3
4
5
6
7
8
9
10
11
12
13
14
15
16
17
18
19
20
21

For peer review only - http://bmjopen.bmj.com/site/about/guidelines.xhtml

For peer review only - http://bmjopen.bmj.com/site/about/guidelines.xhtml

43 - How many nights did you stay in the hospital or birth centre after your baby was born?

0
1
2
3
4
5
6
7
8
9
10

44.a - If yes, how many nights?

1
2
3
4
5
6
7
8
9
10

45 - How many weeks old is your baby TODAY?

1
2
3
4
5
6
7
8
9
10
11
12

For peer review only - http://bmjopen.bmj.com/site/about/guidelines.xhtml

For peer review only

Checklist for Reporting Of Survey Studies (CROSS)

Section/topic	Item	Item description	Reported on page #
**Title and abstract**			
Title and abstract	1a	State the word "survey" along with a commonly used term in title or abstract to introduce the study's design.	P1
	1b	Provide an informative summary in the abstract, covering background, objectives, methods, findings/results, interpretation/discussion, and conclusions.	Abstract
**Introduction**			
Background	2	Provide a background about the rationale of study, what has been previously done, and why this survey is needed.	P1
Purpose/aim	3	Identify specific purposes, aims, goals, or objectives of the study.	P1
**Methods**			
Study design	4	Specify the study design in the methods section with a commonly used term (e.g., cross-sectional or longitudinal).	P1,2
Data collection methods	5a	Describe the questionnaire (e.g., number of sections, number of questions, number and names of instruments used).	P2
	5b	Describe all questionnaire instruments that were used in the survey to measure particular concepts. Report target population, reported validity and reliability information, scoring/classification procedure, and reference links (if any).	P2
	5c	Provide information on pretesting of the questionnaire, if performed (in the article or in an online supplement). Report the method of pretesting, number of times questionnaire was pre-tested, number and demographics of participants used for pretesting, and the level of similarity of demographics between pre-testing participants and sample population.	P2
	5d	Questionnaire if possible, should be fully provided (in the article, or as appendices or as an online supplement).	Appendix
Sample characteristics	6a	Describe the study population (i.e., background, locations, eligibility criteria for participant inclusion in survey, exclusion criteria).	P2
	6b	Describe the sampling techniques used (e.g., single stage or multistage sampling, simple random sampling, stratified sampling, cluster sampling, convenience sampling). Specify the locations of sample participants whenever clustered sampling was applied.	P2
	6c	Provide information on sample size, along with details of sample size calculation.	P2
	6d	Describe how representative the sample is of the study population (or target population if possible), particularly for population-based surveys.	P3,4
Survey	7a	Provide information on modes of questionnaire administration, including the type and number of contacts, the location where the survey was conducted (e.g., outpatient	P2

For peer review only

administration		room or by use of online tools, such as SurveyMonkey).	
	7b	Provide information of survey's time frame, such as periods of recruitment, exposure, and follow-up days.	P2
	7c	Provide information on the entry process:	P3
		–>For non-web-based surveys, provide approaches to minimize human error in data entry.	P3
		–>For web-based surveys, provide approaches to prevent "multiple participation" of participants.	P3
Study preparation	8	Describe any preparation process before conducting the survey (e.g., interviewers' training process, advertising the survey).	P2
Ethical considerations	9a	Provide information on ethical approval for the survey if obtained, including informed consent, institutional review board [IRB] approval, Helsinki declaration, and good clinical practice [GCP] declaration (as appropriate).	P3
	9b	Provide information about survey anonymity and confidentiality and describe what mechanisms were used to protect unauthorized access.	P3
Statistical analysis	10a	Describe statistical methods and analytical approach. Report the statistical software that was used for data analysis.	P3
	10b	Report any modification of variables used in the analysis, along with reference (if available).	N/A
	10c	Report details about how missing data was handled. Include rate of missing items, missing data mechanism (i.e., missing completely at random [MCAR], missing at random [MAR] or missing not at random [MNAR]) and methods used to deal with missing data (e.g., multiple imputation).	Througho ut findings section
	10d	State how non-response error was addressed.	N/A<br>P12
	10e	For longitudinal surveys, state how loss to follow-up was addressed.	N/A
	10f	Indicate whether any methods such as weighting of items or propensity scores have been used to adjust for non-representativeness of the sample.	N/A
	10g	Describe any sensitivity analysis conducted.	N/A
**Results**			
Respondent characteristics	11a	Report numbers of individuals at each stage of the study. Consider using a flow diagram, if possible.	N/A
	11b	Provide reasons for non-participation at each stage, if possible.	N/A
	11c	Report response rate, present the definition of response rate or the formula used to calculate response rate.	N/A p12

For peer review only

	11d	Provide information to define how unique visitors are determined. Report number of unique visitors along with relevant proportions (e.g., view proportion, participation proportion, completion proportion).	N/A
Descriptive results	12	Provide characteristics of study participants, as well as information on potential confounders and assessed outcomes.	P4
	13a	Give unadjusted estimates and, if applicable, confounder-adjusted estimates along with 95% confidence intervals and p-values.	N/A
Main findings	13b	For multivariable analysis, provide information on the model building process, model fit statistics, and model assumptions (as appropriate).	N/A
	13c	Provide details about any sensitivity analysis performed. If there are considerable amount of missing data, report sensitivity analyses comparing the results of complete cases with that of the imputed dataset (if possible).	N/A
**Discussion**			
Limitations	14	Discuss the limitations of the study, considering sources of potential biases and imprecisions, such as non-representativeness of sample, study design, important uncontrolled confounders.	P12
Interpretations	15	Give a cautious overall interpretation of results, based on potential biases and imprecisions and suggest areas for future research.	P11 & p12
Generalizability	16	Discuss the external validity of the results.	P12 N/A
**Other sections**			
Role of funding source	17	State whether any funding organization has had any roles in the survey's design, implementation, and analysis.	Submssion declaration
Conflict of interest	18	Declare any potential conflict of interest.	Submssion declaration
Acknowledgements	19	Provide names of organizations/persons that are acknowledged along with their contribution to the research.	Submssion declaration

For peer review only

BMJ Open

Experience of induction of labour: a cross-sectional postnatal survey of women at UK maternity units

Journal:	*BMJ Open*
Manuscript ID	bmjopen-2023-071703.R1
Article Type:	Original research
Date Submitted by the Author:	28-Mar-2023
Complete List of Authors:	Harkness, Mairi; University of Stirling, NMAHP Research Unit<br>Yuill, Cassandra; City University of London, Centre for maternal and child health research<br>Cheyne, Helen; Nursing Midwifery and Allied Health Professions Research Unit, University of Stirling<br>McCourt, Christine; Department of Midwifery and Child health, City University London<br>Black, Mairead; Aberdeen Maternity Hospital, Aberdeen centre for women's health research<br>Pasupathy, Dharmintra; The University of Sydney<br>Sanders, Julia; Cardiff University, School of healthcare sciences<br>Heera, Neelam; Cysters<br>Wallace, Chlorice; Princess Royal University Hospital<br>Stock, Sarah; The University of Edinburgh Usher Institute of Population Health Sciences and Informatics; The University of Edinburgh MRC Centre for Reproductive Health
**Primary Subject Heading**:	Obstetrics and gynaecology
Secondary Subject Heading:	Health services research
Keywords:	Decision Making, OBSTETRICS, Quality in health care < HEALTH SERVICES ADMINISTRATION & MANAGEMENT

SCHOLARONE™
Manuscripts

I, the Submitting Author has the right to grant and does grant on behalf of all authors of the Work (as defined in the below author licence), an exclusive licence and/or a non-exclusive licence for contributions from authors who are: i) UK Crown employees; ii) where BMJ has agreed a CC-BY licence shall apply, and/or iii) in accordance with the terms applicable for US Federal Government officers or employees acting as part of their official duties; on a worldwide, perpetual, irrevocable, royalty-free basis to BMJ Publishing Group Ltd ("BMJ") its licensees and where the relevant Journal is co-owned by BMJ to the co-owners of the Journal, to publish the Work in this journal and any other BMJ products and to exploit all rights, as set out in our licence.

The Submitting Author accepts and understands that any supply made under these terms is made by BMJ to the Submitting Author unless you are acting as an employee on behalf of your employer or a postgraduate student of an affiliated institution which is paying any applicable article publishing charge ("APC") for Open Access articles. Where the Submitting Author wishes to make the Work available on an Open Access basis (and intends to pay the relevant APC), the terms of reuse of such Open Access shall be governed by a Creative Commons licence – details of these licences and which Creative Commons licence will apply to this Work are set out in our licence referred to above.

Other than as permitted in any relevant BMJ Author's Self Archiving Policies, I confirm this Work has not been accepted for publication elsewhere, is not being considered for publication elsewhere and does not duplicate material already published. I confirm all authors consent to publication of this Work and authorise the granting of this licence.

For peer review only

Title Page

Title

Experience of induction of labour: a cross-sectional postnatal survey of women at UK maternity units

Authors

Dr Mairi Harkness; Dr Cassandra Yuill; Professor Helen Cheyne; Professor Christine McCourt; Dr Mairead Black; Professor Dharmintra Pasupathy; Professor Julia Sanders; Neelam Heera-Shergill; Chloe Wallace; Professor Sarah Stock

Corresponding Author

Dr Mairi Harkness, NMAHP Research Unit, Pathfoot Building, University of Stirling, Stirling, FK9 4LA. Email: mairi.harkness@stir.ac.uk; Phone: 01786 466341.

Author Details

Dr Mairi Harkness, Nursing Midwifery and Allied Health Professionals Research Unit (NMHAP-RU), University of Stirling, Stirling, UK.

Dr Cassandra Yuill, Centre for Maternal and Child Health Sciences, City, University of London, London, UK.

Professor Helen Cheyne, Nursing Midwifery and Allied Health Professionals Research Unit (NMHAP-RU), University of Stirling, Stirling, UK.

Professor Christine McCourt, Centre for Maternal and Child Health Sciences, City, University of London, London, UK.

Dr Mairead Black, Aberdeen Maternity Hospital, Aberdeen, UK.

Professor Dharmintra Pasupathy, University of Sydney, Sydney, Australia

Professor Julia Sanders, School of Healthcare Sciences, University of Cardiff, Cardiff, UK

Chloe Wallace, Princess Royal University Hospital, Orpington, UK

Neelam Heera-Shergill, Cysters (CIO), Birmingham, UK

Professor Sarah J Stock, Usher Institute, and MRC Centre for Reproductive Health, University of Edinburgh, Edinburgh, UK

Key Words

Pregnant women
Peripartum period
Maternal health services
Satisfaction; Patient/labour; Induced; Cervical ripening; Pregnant women; Maternal health services

For peer review only - http://bmjopen.bmj.com/site/about/guidelines.xhtml

Word Count

4589

Footnote: The words woman and women are used throughout this piece however it is important to acknowledge that not all people who have induction of labour identify as women.

Abstract

Objectives: This study explored women's views and experiences of key elements of the induction of labour (IOL) process, including at home or in hospital cervical ripening (CR).

Design: A questionnaire-based postnatal survey undertaken as part of the CHOICE Study process evaluation. The questionnaire was administered online and included fixed response and free text options.

Setting: NHS maternity units in the United Kingdom

Participants: 309 women who had an IOL

Outcome measures: The primary outcome measure was experience of IOL. Few women returned home during CR, meaning that statistical comparison between those who experienced home and hospital based CR was not possible. Findings are reported as descriptive statistics with content analysis of women's comments providing context.

Findings: Information to support choice and understand what to expect about IOL is often inadequate or unavailable. Having IOL can create anxiety and remove options for birth that women had hoped would enhance their experience. Although it can provide a more comfortable environment, home CR is not always an acceptable solution. Women described maternity care negatively impacted by staffing shortages; delays to care sometimes led to unsafe situations. Women who had a positive experience of IOL described supportive interaction with staff as a significant contribution to that.

Conclusions: Women do not experience IOL as a benign and consequence free intervention. There is urgent need for research to better target IOL and optimise safety and experience for women and their babies. Relatively few women were offered CR at home and further research is needed on this experience.

For peer review only

Strengths and Limitations

- A robustly designed survey, including use of previously tested tools, was used to determine key aspects of women's experience of induction of labour.
- Carefully considered recruitment strategies resulted in a large sample across multiple NHS sites.
- Few women returned home during cervical ripening. As a result data analysis produced descriptive, rather than inferential, statistics.
- Qualitative analysis of women's free text comments adds important context and aids understanding and interpretation of the findings.

For peer review only - http://bmjopen.bmj.com/site/about/guidelines.xhtml

- The survey was conducted during the COVID-19 pandemic and findings should be considered within this unique context.

Manuscript

Background

Globally, induction of labour (IOL) rates have increased steadily over the last 20 years, with a recent surge in rates linked to improved evidence of safety and efficacy (1). In the UK around 30-50% of births currently involve IOL, making it one of the most common obstetric interventions (2,3).

Impact of IOL on women's experience of childbirth is unclear. Some evidence suggests that IOl has little effect on overall satisfaction when compared to spontaneous labour (1), however, undergoing IOL is understood to affect experience of childbirth: it is generally more painful than spontaneous labour; more likely to lead to additional interventions such as operative birth and epidural analgesia; and may remove the satisfaction of experiencing the more natural birth that many hope for (4,5,6).

A positive birth experience is not merely nice to have. Women's experience during childbirth is described by the World Health Organisation (WHO) as a 'critical aspect of ensuring high-quality labour and childbirth care (7). Evidence underpins socio-cultural and psychological aspects of care as significant for women during childbirth (8), and negative experience of childbirth can be linked to serious psychological harm (9). Despite this there remains a dearth of evidence about women's experience of IOL.

The first stage of IOL, cervical ripening (CR), involves application of a drug or mechanical method to change a woman's cervix in preparation for labour. The second phase, if labour onset does not occur as a result of CR alone, is artificial rupture of the fetal membranes and intravenous administration of oxytocin. Traditionally the whole process of IOL has been undertaken in hospital, however, some maternity units now offer home CR: women attend hospital for initial assessment and administration of CR agent and then return home for a period before labour starts or reassessment in hospital. Some evidence indicates that home cervical ripening could reduce duration of hospital stay during IOL and improve women's experience. There is increasing evidence to suggest that home CR is safe (10,11,12), although its acceptability to women and impact on staff and maternity service has not been fully evaluated (13).

The aim of this study was to explore women's views of their IOL, with a specific focus on their experiences of the initial stages of the process including CR at home and in-hospital.

For peer review only - http://bmjopen.bmj.com/site/about/guidelines.xhtml

Methods

Design

This study was undertaken as part of the CHOICE Study, a prospective cohort study and process evaluation[8] commissioned by the National Institute for Health and Care Research (NIHR) to examine the safety, efficacy and acceptability of home CR and CR in hospital. The process evaluation (qCHOICE) included a postnatal questionnaire-based survey (reported here).
A cross-sectional online survey was used with the aim of describing women's views and experience of IOL, particularly those having CR at home and in-hospital.

Questionnaire development

The questionnaire was designed to explore key elements of the IOL process, using fixed response and free-text questions. The questionnaire assessed satisfaction, sense of control and mental wellbeing including previously tested tools (14,15).

The IOL satisfaction questionnaire (14) was used to assess women's experiences of IOL, including information provision, anxiety, and physical and emotional discomfort. This questionnaire uses a five-point Likert scale (Strongly agree – strongly disagree), we analysed this using N (%) agreement to create three categories: merging strongly agree with agree, and strongly disagree with disagree.

A series of ten questions from the labour agentry scale (short) (15) were used to measure sense of control during childbirth. We used a six-point Likert type scale, and analysis was reported as percentage agreement across three categories: agree, neutral and disagree.

Demographic questions and questions about information and decision-making were based on those in the Scottish National Maternity Survey (16) altered to focus on IOL. The survey also included questions about cost of CR (home and hospital), including travel and childcare, for health economics evaluation to be reported elsewhere.

The questionnaire was pilot tested with the CHOICE Study personal and public involvement (PPI) group (women with recent experience of childbirth) and with maternal health researchers at City, University of London (11 people in total). Feedback was used to make minor changes to six questions, particularly those concerning decision-making and choice, improving clarity and accessibility. The scale used for the IOL satisfaction scale (14) was changed from "Never; Rarely; Some of the time; Most of the time; Always" to "Strongly agree; Agree; Unsure; Disagree; Strongly disagree".

The survey was online, hosted by *Online Surveys* (www.onlinesurveys.ac.uk); a written version or completion via telephone and/or with a translator could be requested.

Sample and recruitment

A convenience sampling approach was used, with women who underwent IOL at 37 weeks of pregnancy or more at the 21 NHS sites participating in the CHOICE study potentially eligible. Those who experienced pregnancy loss were ineligible.

The planned sample size was calculated to enable comparison of the experiences of women who had home and hospital cervical ripening, as per the CHOICE Study aims (13). The sample size required to compare the experiences of women who had home and hospital cervical ripening is estimated to be

For peer review only - http://bmjopen.bmj.com/site/about/guidelines.xhtml

89 per group (178 in total) for a probability of type 1 error set at 0.05 for a two-tailed comparison and a 80% power. This is based on use of the labour agentry scale (15) where a change of 5.5 points is considered clinically meaningful

The initial recruitment strategy was via electronic maternity notes system BadgerNet. We anticipated that women would receive information about the study using push notifications sent when an IOL was booked, and again at around ten days postnatal when maternity care ended; directing them to view study information on their electronic maternity notes and access the study link. However, initial response rate was poor, it was not clear to what extent push notifications were being received, therefore additional strategies were put in place while the survey remained open: firstly, efforts were targeted to seven sites (the five case study sites plus two that had expressed interest in the survey) to obtain a more focused response. At these sites a research midwife identified eligible participants on the postnatal ward and handed them a study card with a link to the online survey; In addition, a targeted social media advertising campaign (Facebook) was used in the five qCHOICE case study sites.

The questionnaire included initial eligibility screening questions, and ineligible respondents could not proceed to complete the survey.

The survey was open between February 2021 and April 2022. The planned sample calculation was subsequently revised when it became apparent that too few women were having CR at home for statistical comparison to be made. A sample size of 300 respondents was deemed practical, achievable and useful for the purpose of describing the experience of women who undergo CR.

Patient and public involvement statement

The CHOICE Study PPI group were involved in development of participant information materials and survey recruitment strategies. The survey questionnaire was pilot tested with the group. A member of the PPI group is co-author for this paper and will be involved in further dissemination of findings.

Consent for participation

The questionnaire landing page included detailed participant information, researcher contact details for further information, and consent questions to be completed before the survey could be accessed.

Data Analysis

Survey responses were exported from the online survey site into IBM SPSS Statistics 23 software. Data were de-identified, cleaned and statistics produced.

We found that 12% (n=36) of the eligible survey respondents returned home during CR therefore it was not possible to use inferential statistics to compare their experience with those who remained in hospital. Instead, descriptive analysis was used across both groups with analysis of free text responses providing context to the women's experience.

Free-text responses were analysed using thematic analysis approach; determining themes and, for some questions, how often those themes occurred. Initial analysis was undertaken by a single

For peer review only - http://bmjopen.bmj.com/site/about/guidelines.xhtml

researcher, with emerging themes discussed and confirmed with three further members of the qCHOICE team, in an iterative process. NVivo 12 software was used to organise the data and assist analysis.

Findings

In total 320 questionnaires were completed. Nine responses were excluded as respondents had not had an IOL and a further two because their IOL happened prior to the CHOICE Study commencing. Three hundred and nine eligible responses were included in the analysis.

Study Respondents

Respondents had given birth in Scotland, England and Wales at 19 CHOICE Study sites and a further six NHS areas. Descriptive data for those who responded are given in Table 1.

For peer review only

Table 1: Description of the survey respondents

First baby?<br>N=309	Yes:<br>206 (67%)	No:<br>103 (33%)			
Maternal Age (years)<br>N=309	Min: 19	Max: 52	Median: 31<br>St. deviation: 4.993		

For peer review only - http://bmjopen.bmj.com/site/about/guidelines.xhtml

			Variance: 24.932		
Ethnicity N=307 (2 missing)	White 291 (95%)	Asian/Asian British 8 (3%)	Black: 4 (1%)	Mixed/multiple ethnicity: 4 (1%)	
Social deprivation index* N=306 (3 missing)	1 (most deprived) 61 (20%)	2 57 (19%)	3 60 (20%)	4 73 (24%)	4 (least deprived) 55 (18%)
Baby's birthweight (grams) N=297 (12 missing)	Min: 1790	Max: 6600	Median: 3500		
Gestation at IOL (weeks) N=309	Min:37	Max: 42	Median: 39		
Reason for IOL N=309	Medical (eg raised blood pressure) 146 (47%)	Post dates 70 (23%)	Size of baby (large or small) 37 (12%)	Spontaneous rupture of membranes (SROM) 20 (7%)	Reduced fetal movements (RFM) 19 (6%)
Cervical ripening? N=304 (5 missing)	Yes 266 (86%)	No 38 (12%)			
Method of induction of labour N=309	Prostaglandin gel/pessary 202 (65%)	Balloon catheter 43 (14%)	Non-CR methods: membrane sweep, amniotomy, intravenous oxytocic 38 (12%)	Prostaglandin gel/pessary and balloon catheter 12 (4%)	Osmotic dilator (eg. Dilapan-S) 9 (3%)
Home cervical ripening N=266	Offered option to return home 39 (15%)	Returned home 36 (13%)			

*Social deprivation index quintiles: Scottish Index of Multiple Deprivation and English Indices of Deprivation (based on self-reported postcode).

Decision making

The questionnaire included a series of questions about choice, decision-making and provision of information when offered IOL (Table 2). Fifty seven percent of women reported that they had either

For peer review only - http://bmjopen.bmj.com/site/about/guidelines.xhtml

no choice, or no alternative option, about having IOL. While two thirds (66%) felt that options were explained in a way they could understand, only half (50%) felt that they fully understood what to expect during IOL.

Table 2: Choice, decision making and information

**Did you feel you were offered a choice about having your labour induced or waiting for labour to start?**	
Yes, I felt it was fully my decision	122 (39%)
Yes, but I felt there was no other option	117 (38%)
Not really, as I didn't have enough information	10 (3%)
No, I didn't feel I was given a choice	60 (19%)
**Were these options explained to you in a way that you could understand?**	
Yes, I felt I fully understood	205 (66%)
Partly	70 (23%)
Not really	19 (6%)
I'm not sure	2 (0.6%)
No	13 (4%)
**Did you get enough information about what to expect during induction of labour?**	
Yes, I felt I fully understood	155 (50%)
Partly	90 (29%)
Not really	38 (12%)
I'm not sure	0
No	26 (8%)
**Did having an induction lead to any change in your birthplace plans?**	
Yes	148 (48%)
No	153 (59%)
I'm not sure	8 (3%)

The free text responses describe anxiety around risk to their baby's wellbeing as a major influence on the decision to accept the offer of an IOL. For some women this risk was communicated in a way that that contributed to them feeling their choice about IOL was limited.

"I was induced because of my age. Whilst it was made clear that the decision was my choice, I also felt a lot of pressure from health professionals to be induced" {Participant 010, Multip, Hospital CR}

"It was never something I had a choice in… I was told if I didn't get induced there was a high chance of my baby being stillborn because I was almost 42 weeks, so this scared me." {Participant 201, Primip, Home CR}

One hundred and forty-eight (48%) respondents stated that having an IOL changed their plans for labour and birth. Changes included: unable to use water immersion; change of planned place of birth to an obstetric unit from midwifery led unit (MLU) or home birth. Women also reported needing previously unwanted interventions including electronic fetal monitoring and intravenous oxytocin

For peer review only - http://bmjopen.bmj.com/site/about/guidelines.xhtml

"I would have liked a water birth but was told it was no longer an option" {Participant 080, Primip, Hospital CR}

Time spent in hospital and at home during cervical ripening

Of the 266 respondents who had CR 39 (15%) were given the option to return home and 36 (14%) did return home. Of those, 22 (61%) had their IOL at a single maternity unit where home CR was offered to all women unless contraindicated.

Some women expressed disappointment at not having the option to return home, whereas others would not have wanted this option. Common themes were lack of choice about where CR occurred and feeling safer in hospital.

"I was told I could have balloon induction and go home at consultant appointment, then when I attended hospital was told this wasn't actually something I could have." {Participant 100, Primip, Hospital CR}

"I am pleased I didn't have a choice {of home CR} and stayed overnight. I did have a comfort I was in the right place." {Participant 081, Primip, Hospital CR}

For women who remained in hospital, the median duration in the antenatal area, between commencing CR and transfer to labour suite, was 22 hours. One hundred women (43%) reported being in antenatal area for 24 hours or longer and 42 (18%) for 48 hours or more. The longest duration of antenatal stay after CR commenced until transfer to a labour room was 260 hours; 11 days. Those who returned home remained at home for a median of 24hours (range: 3 to 168 hours).

The respondents described delays at almost every stage of the IOL process. The most impactful was the wait to be transferred from antenatal area to labour ward after CR, either for artificial rupture of the fetal membranes (ARM) or because they were in labour. Staffing was frequently mentioned in relation to delays.

"The staff were pushed to the brink which is why I was in hospital for 11 days before my waters were broken." {Participant 120, Primip, Hospital CR}

For some women the delay between the decision being made for IOL and the process being started, and subsequent delays after IOL commenced, conflicted with the information that their baby was at risk of death if the pregnancy continued and needed to be born soon.

For peer review only - http://bmjopen.bmj.com/site/about/guidelines.xhtml

"When you have been told for 3 months that your baby could be in danger if you reach 39 weeks and then have to go beyond that because they don't have a bed for you, it's a very scary time" {Participant 080, Primip, Hospital CR}

Some women described care being planned around service capacity rather than in line with guidance. At times this was described as having a direct effect on their IOL progress.

"Had balloon induction 8am Monday. Balloon out 8am Tuesday and was 2-3cm. However was sent home as there were not enough midwives to induce me further… Was taken back in on Thursday 4pm… 7am Friday taken to the delivery room… at that point was then back to 1cm." {Participant 194, Primip, Home CR}

Women often described poor experience of time spent in antenatal areas during CR: lack of privacy, lack of sleep, lack of food. They also reported a shortfall in support that midwives were able to provide before transfer to labour suite, manifested in lack of appropriate pain relief, lack of emotional support and concerns about clinical care.

"I was labouring behind a curtain, no privacy, others all around me… It was really hard to focus and stay calm and relax with no privacy of my own, no pain relief and no food." {Participant 036, Multip, Hospital CR}

"I spent 3 days crying in pain unable to eat or sleep in hospital" {Participant 135, Primip, Hospital CR}

Among women who stayed in hospital throughout the induction process 196 (74%) had a birth partner who stayed with them compared to 40 (98%) women who returned home. Free text responses indictated that when CR happened in hospital birth partners were not always able to stay as often as women wanted.

"It was a lonely experience, my husband was not allowed to come in until I was in active labour" {Participant 137, Multip, Hospital CR}

Presence of their birth partner was very important to the respondents, with their absence described as absence of important support. It was also reported that exclusion of birth partners, usually the other parent, denied them full participation in an important life experience.

"the induction also meant my husband actually missed our son being born because I progressed so quickly" {Participant 225, Multip, Hospital CR}

"My partner and I feel like one of the most important experiences of our lives was stolen from us" {Participant 264, Primip, Hospital CR}

For peer review only - http://bmjopen.bmj.com/site/about/guidelines.xhtml

Twenty-eight women described being in established labour for a prolonged period and/or approaching second stage while remaining in the antenatal area.

"I was told I couldn't have [epidural analgesia l] until I moved to labour ward but I couldn't move to labour ward as it was full. I was only moved when I was pushing" {Participant 113, Primip, Hospital CR}

Experience of labour induction

The findings suggest that for many women IOL, regardless of any time spent at home, is a period of anxiety, pain and discomfort, and of feeling powerless and lacking control (Table 3). Over a third of women (101) (38% who remained in hospital and 44% of those who went home) did not feel comfortable with their decision to have an IOL, whilst 22% (21% who remained in hospital and 31% who returned home) were worried that IOL might not be safe. Although 36% of those who went home reported anxiety about this, in relation to future choice of home or hospital for CR, more than half in each case (55% hospital and 64% home) said they would choose the same option again.

While 67% of women who stayed in hospital reported having good family support throughout the induction, this was 97% for those who went home.

Table 3: Satisfaction during CR and IOL (Henry et al, 2013)

**From initiation of CR to admission to Labour suite**	**Women who remained in hospital N=227 (3 missing)**		**Women who returned home N=36 (0 missing)**	
	**Agree**	**Unsure & Disagree**	**Agree**	**Unsure & Disagree**
I felt a lot of discomfort	143 (63%)	84 (37%)	28 (78%)	8 (22%)
I was able to cope with the discomfort	155 (68%)	72 (32%)	29 (81%)	7 (19%)
I felt anxious about being in hospital /going home	115 (51%)	112 (49%)	14 (39%)	22 (61%)
I was able to relax on the AN ward/ at home	101 (44%)	126 (56%)	20 (56%)	16 (44%)
I was able to rest on the AN ward/home	103 (45%)	124 (55%)	24 (67%)	12 (33%)
I had good family support in hospital/home	151 (67%)	76 (33%)	35 (97%)	1 (3%)
I had easy access to information from the staff	127 (56%)	100 (44%)	23 (64%)	13 (36%)

For peer review only - http://bmjopen.bmj.com/site/about/guidelines.xhtml

I was worried the induction might not be safe	47 (21%)	180 (79%)	11 (31%)	25 (69%)
I would have preferred to go home/stay at the hospital	97 (43%)	130 (57%)	12 (33%)	24 (67%)
I felt embarrassed by the catheter or gel	21 (9%)	206 (91%)	3 (8%)	33 (92%)
While at home I felt anxious about being at home not hospital	N/A	N/A	13 (36%)	23 (64%)
**IOL**	N= 230		N=36	
I felt anxious about being induced	168 (73%)	62 (26%)	31 (86%)	5 (14%)
I felt in control	62 (27%)	168 (73%)	9 (25%)	27 (75%)
I understood what was happening	174 (76%)	56 (24%)	24 (67%)	12 (33%)
I felt relaxed	62 (27%)	168 (72%)	8 (22%)	28 (78%)
Everything made sense	137 (60%)	93 (40%)	18 (50%)	18 (50%)
I was given clear information	151 (66%)	79 (34%)	17 (47%)	19 (53%)
I felt comfortable with my choice about my care	145 (63%)	85 (37%)	20 (56%)	16 (44%)
I had access to information about the types of induction available	119 (52%)	111 (48%)	20 (56%)	16 (45%)
I had easy access to information about what to do	122 (53%)	108 (47%)	19 (53%)	17 (48%)
I found the induction process uncomfortable	144 (63%)	86 (38%)	32 (89%)	4 (11%)
I was worried about when my labour would begin	176 (76%)	54 (22%)	26 (72%)	10 (28%)
I would choose staying in hospital /going home again	126 (55%)	104 (45%)	23 (64%)	13 (36%)
I would recommend staying in hospital during induction/going home to other women	125 (54%)	105 (36%)	22 (61%)	14 (39%)

Findings related to aspects of participants sense of control are described (Table 4). Overall around one third of women reported feeling like a failure, 40% felt powerless; about half felt fearful and around half felt confident or in control. Most women (both home and hospital) reported feeling that they were with people who cared about them.

Table 4: Sense of Control during induction of labour

For peer review only - http://bmjopen.bmj.com/site/about/guidelines.xhtml

	**Agree All respondents N=309**	**Agree In-hospital CR N=230**	**Agree Home CR N=36**
I felt tense	188 (61%)	143 (62%)	23 (64%)
I felt important	215 (69%)	149 (65%)	28 (78%)
I felt confident	149 (48%)	102 (44%)	19 (53%)
I felt in control	140 (45%)	98 (43%)	18 (50%)
I felt fearful	152 (49%)	113 (49%)	19 (53%)
I felt relaxed	95 (31%)	68 (30%)	10 (28%)
I felt good about my behaviour	243 (79%)	183 (80%)	27 (75%)
I felt helpless (powerless)	122 (39%)	92 (40%)	15 (42%)
I felt like a failure	96 (31%)	71 (31%)	12 (33%)
I felt I was with people who care about me	249 (81%)	179 (78%)	31 (86%)

Respondents' feelings of anxiety, powerlessness and lack of control were apparent in free text comments:

"I felt like things happened to me rather than being part of any decisions" {Participant 109, Primip, Hospital CR}

"I felt that choices were taken away from me… I don't think I was given enough information… I wasn't told whether this [painful CR] was normal" {Participant 113, Primip, Hospital CR}

Forty-one women described their experiences of IOL as traumatic and/or having caused significant long-term negative impact on their physical and/or mental wellbeing.

"It was all so horrendous that I will never have another child. It gives me anxiety thinking about it all. Before this experience I did want more than one child" {Participant 184, Primip, Hospital CR}

Thirty-five women described experiences that were positive overall. Supportive interaction with staff made a significant contribution to women's positive experiences, as did feeling 'safe' and 'cared for'.

"Supportive staff, well informed and felt every decision was genuinely done for our wellbeing" {Participant 092, Primip, Hospital CR}

"My midwife {name} was incredibly supportive throughout labour and birth. We felt safe and cared for." {Participant 040, Primip, Hospital CR}

For peer review only - http://bmjopen.bmj.com/site/about/guidelines.xhtml

For peer review only

Discussion

This study reports on the experience of women undergoing CR and IOL more generally, describing wide variation. While some women report a positive experience, significant numbers described a negative experience and a small but important number had an experience that was so traumatic they wished to avoid future, previously planned, pregnancies.

The IOL process begins when a pregnant woman and her care giver first discuss IOL; facilitation of informed decision-making is integral to quality maternity care and prominent within current NICE guidance around IOL (4). However, decision making about IOL may be complex and for many of the women in this survey it seemed poorly supported: most respondents (60%) felt that they either had no choice about IOL or no alternative option. Communicating risk in relation to IOL can be difficult and contentious (17), and this study found that communication around IOL led some women to believe that induction was required to avoid an otherwise high chance of their baby dying. Informed decision making must be underpinned by good quality information, and clinicians should include absolute as well as relative risks of stillbirth when sharing information with women.

Provision of antenatal education and information are recognised as key factors in shaping women's expectations and their ability to cope with labour and birth (18). However, just half (50%) of the survey respondents felt that they fully understood what to expect during their IOL and almost a third (32%) felt unable to cope with the discomfort of CR. Active decision-making may contribute to positive experience when women require previously unwanted interventions (19), however, the women surveyed here described an absence of real choice about IOL alongside significant restrictions on options for care when they accept induction. The most reported restrictions on birth plans were accessing an MLU and use of water during labour or birth. Both are known to improve experience: water is an effective method of pain relief during the first stage of labour (4); births planned in MLUs are associated with significantly reduced intrapartum interventions, with no difference in neonatal outcome (20,21) and increased satisfaction with care (22,23).

Free text responses reveal further impact on experience of care, with women describing a paradox of deciding, or sometimes being persuaded, to have an IOL because of perceived risk but then facing an absence of urgency to commence induction and significant delays during the process itself. This was a marked aspect of negative experience and caused stress and anxiety, especially when women reported having been told they required induction because their baby was at risk of stillbirth.

Few women were given the option to return home after CR commenced, limiting our ability to explore whether home CR may improve experience. Of the small number (just 36 women) who did more than half would recommend this option, most were able to cope with the discomfort they felt and most felt able to rest and relax during CR. This is despite most also having had CR using mechanical methods which may be associated with increased initial pain (6, 14). Nonetheless, a third of women who went home would have preferred to stay in hospital and 36% felt anxious about being at home rather than in hospital.

Those who experienced CR in hospital describe an environment that frequently failed to meet their needs. Time spent in hospital, during CR and prior to transfer to labour ward, was often characterised by inadequate support from staff and absence of birth partners, combined with insufficient pain relief, lack of privacy in shared wards and failure to take seriously or listen to women's concerns about pain, discomfort and labour progress.

For peer review only - http://bmjopen.bmj.com/site/about/guidelines.xhtml

Delays were reported at almost every stage of the IOL process, the most impactful being late assessment of progress and application of further CR agent, and long delays when ready for transfer to labour ward. The women associated delays with poor staffing, reflecting similar recent experience of UK maternity services (24,25). It was not unusual for respondents to feel that their physical safety was compromised; some reporting eventual transfers to delivery suite during advanced labour including second stage. Thus, women who had been informed their baby was at increased risk of death were receiving care below that required once in established labour.

Physical safety and psychological wellbeing are equally valued by women (19) and some respondents reported that they felt the care they received compromised both. Mental ill health is a leading and increasing cause of maternal morbidity and mortality (26). That around half the women (49%) in this survey reported feeling fearful during their IOL, and that many described an experience that was traumatic with lasting negative impact, is of significant concern.

It is of note that there is extensive literature on women's negative experience but much less on the nature of women's positive experience (8). Some women who responded to the survey did have positive experiences of IOL, and the features they describe offer insight to aid understanding of how best to support women undergoing IOL. The most significant factor in women's positive experience was their interaction with staff. This echoes longstanding knowledge of the importance interaction with care givers holds for women's experience (18), and is something that individual practitioners can influence despite organisational and workforce factors.

The overwhelming majority of respondents (81%) stated that they felt that they were with someone who cared about them, but concerningly, nearly 20% of women did not feel this way. Compassionate and respectful care encompasses a sense of care as genuine through 'emotional availability' (8) and it is encouraging that this appears to have been facilitated for most. For many women enabling birth partner attendance during the difficult and often lengthy period of CR would be a simple and effective means of further supporting this.

Limitations

The sampling and recruitment strategies employed meant that it was not possible to determine a denominator from which a response rate could be calculated. It is recognised that this may introduce bias among the characteristics and experiences of those who chose to respond. However, adoption of a pragmatic approach to achieve a large sample across multiple NHS sites was deemed to outweigh potential limitations. The results are descriptive and not intended to be generalisable.

The COVID-19 pandemic presented significant challenges, both to study recruitment and to the context in which the respondents received the maternity care they were describing. Maternity unit policies and practices changed in response to various stages of the pandemic, with at least one of the five case study sites halting the offer of home IOL completely. Some placed severe restrictions on the presence of partners on antenatal wards, although it is of note that pre-pandemic it was often usual practice to restrict birth partner attendance to visiting hours only. The survey findings should be understood within this context.

The work was undertaken in the UK at a time when NHS maternity services were under significant strain, and experiencing a significant rise in induction rates. While there is no doubt that this context impacts the findings and their interpretation, it was clear that factors unrelated to staff shortages were also influencing the experiences reported here. In addition, findings such as lack of informed choice, pain and anxiety, fear and concerns about lack of monitoring or support until in active labour have been identified in studies conducted prior to current staff shortages (6). Similar difficulties also

For peer review only - http://bmjopen.bmj.com/site/about/guidelines.xhtml

impact provision of maternity services in many countries worldwide and, without significant systemic and economic change, is likely to remain the context in which IOL is offered for the immediate future.

A significant limitation was that so few women were offered the option to return home during CR (n=36, 12%), limiting the opportunity to compare their experience with those who remained in hospital. However, there is very little information about women's experience of home CR and this work adds important and relevant understanding about women's experience of undergoing IOL, cervical ripening in particular, both in hospital and at home.

Conclusion

This work shows that women undergoing IOL do not experience it as a benign, consequence free intervention; induction of labour often causes anxiety and removes options for birth that women had hoped would enhance their experience and outcomes. Assessing risks and benefits when offering, and considering the offer of, an IOL can be complex; clinicians must ensure that women are informed of and understand the absolute as well as relative risks of continuing the pregnancy, along with the risks and consequences of IOL itself.

Experience of childbirth is important to women; known to influence physical, social and psychological short and long-term outcomes. Although some women had a positive experience of IOL, many experienced poor care, inadequate communication and delays during IOL that had the potential to jeopardise their safety or that of their baby. For some, this led to long term psychological maternal morbidity including the desire to avoid future pregnancies.

Returning home during CR may be an option to improve women's experience of IOL; however few women were offered this opportunity and numbers were too small to draw definite conclusions.

Women's experience of childbirth was profoundly affected by staffing and resource issues; this context makes it difficult to extrapolate poor experience of IOL from poor experience of childbirth due to lack of staff and subsequent inadequate care.

Induction of labour rates have been increased with good intentions of reducing rates of stillbirth and severe neonatal morbidity, but this does not always appear to have been accompanied by adequate planning or increase in the facilities and resources that are necessary to provide an effective service. In addition, the findings of this study indicate that greater attention to the quality of information giving to underpin informed choice is needed. This accords with a recent report on risk assessment in maternity pathways in the UK, which calls for more individualised risk assessments (27).

It is crucial that the expected principles of person-centred individualised care, provided with dignity and respect, apply equally to women experiencing IOL. Listening to women and service users, ensuring that practice is based on their needs, and that services are sufficiently resourced are all essential to the provision of safe and effective induction pathways.

For peer review only

For peer review only - http://bmjopen.bmj.com/site/about/guidelines.xhtml

Author Contribution Statement

Dr Mairi Harkness: Substantial contribution to the design of the work, and acquisition, analysis, and interpretation of data for the work. Drafted the work and revised it critically for important intellectual content. Final approval of the version to be published. Agrees to be accountable for all aspects of the work in ensuring that questions related to the accuracy or integrity of any part of the work are appropriately investigated and resolved.

Dr Cassandra Yuill: Substantial contribution to the design of the work, and acquisition, analysis, and interpretation of data for the work. Revised the work critically for important intellectual content. Final approval of the version to be published. Agrees to be accountable for all aspects of the work in ensuring that questions related to the accuracy or integrity of any part of the work are appropriately investigated and resolved.

Professor Helen Cheyne: Substantial contribution to the design of the work, and acquisition, analysis, and interpretation of data for the work. Revised the work critically for important intellectual content. Final approval of the version to be published. Agrees to be accountable for all aspects of the work in ensuring that questions related to the accuracy or integrity of any part of the work are appropriately investigated and resolved.

Professor Christine McCourt: Substantial contribution to the design of the work, and acquisition, analysis, and interpretation of data for the work. Revised the work critically for important intellectual content. Final approval of the version to be published. Agrees to be accountable for all aspects of the work in ensuring that questions related to the accuracy or integrity of any part of the work are appropriately investigated and resolved.

Dr Mairead Black: Substantial contribution to the conception and design of the work, and interpretation of data for the work. Revised the work critically for important intellectual content. Final approval of the version to be published. Agrees to be accountable for all aspects of the work in ensuring that questions related to the accuracy or integrity of any part of the work are appropriately investigated and resolved.

Professor Dharmintra Pasupathy: Substantial contribution to the conception and design of the work. Revised the work critically for important intellectual content. Final approval of the version to be published. Agrees to be accountable for all aspects of the work in ensuring that questions related to the accuracy or integrity of any part of the work are appropriately investigated and resolved.

Professor Julia Sanders: Substantial contribution to the conception and design of the work, and interpretation of the data for the work. Revised the work critically for important intellectual content. Final approval of the version to be published. Agrees to be accountable for all aspects of the work in ensuring that questions related to the accuracy or integrity of any part of the work are appropriately investigated and resolved.

Chloe Wallace: Substantial contribution to the design of the work. Revised the work critically for important intellectual content. Final approval of the version to be published. Agrees to be accountable for all aspects of the work in ensuring that questions related to the accuracy or integrity of any part of the work are appropriately investigated and resolved.

For peer review only - http://bmjopen.bmj.com/site/about/guidelines.xhtml

Neelam Heera-Shergill: Substantial contribution to the design of the work. Revised the work critically for important intellectual content. Final approval of the version to be published. Agrees to be accountable for all aspects of the work in ensuring that questions related to the accuracy or integrity of any part of the work are appropriately investigated and resolved.

Professor Sarah Stock: Substantial contribution to the conception and design of the work, and interpretation of data for the work. Revised the work critically for important intellectual content. Final approval of the version to be published. Agrees to be accountable for all aspects of the work in ensuring that questions related to the accuracy or integrity of any part of the work are appropriately investigated and resolved.

Competing Interests Statement

The authors declare the following competing interests

Support for the present manuscript: The CHOICE Study was funded by National Institute of Healthcare Research Health Technology and Assessment (NIHR HTA). The grant was paid to institution and the authors have the following associations: Patient and public involvement (PPI) Group member (paid); Funded research fellows (2); Principal investigator and co-investigators with funded time.

CHOICE study budget has been used by some authors for travel and conference cost.

Authors hold additional, paid to institution, grants from: NIHR; Wellcome Trust; Medical Research Council; Chief Scientist Office of Scotland; Tommy's Charity; Scottish government; Aberlour Childcare Trust (small project grant); University of Stirling article processing fund.

Authors declare the following: Consultancy fees: Natera, consultancy on pre-term birth treatments (paid to institution); Honoria: Hologic, Honoria for educational talk (paid to institution); Expert testimony: Expert witness in (midwifery) in civil litigation claims (self employed)

Authors declare the following participation on data safety monitoring or advisory boards: Membership of several NIHR Trial Steering committees; NIHR Health Technology and Assessment DMC and TSC

Authors are unpaid members or trustees of the following Boards and Committees: National Institute of Health and Care Excellence Antenatal Guideline Group 2018-202; Cysters (CIO) Chair of trustees; Platform Housing Group, trainee Board role; UK Government Period Poverty Task Force, member; UK sepsis Trust, Trustee

Funding Statement

The CHOICE Study was funded by the National Institute of Healthcare Research Health Technology and Assessment (NIHR HTA) NIHR127569. The views expressed are those of the authors and not necessarily those of the NIHR or the Department of Health and Social Care.

For peer review only - http://bmjopen.bmj.com/site/about/guidelines.xhtml

Data sharing statement

The data sets generated during this study are available from the corresponding author upon reasonable request.

Ethics Approval

Ethics approval was obtained from the York & Humber – Sheffield Research Ethics Committee in June 2020 (IRAS: 276788) as part of the CHOICE Study application.

Acknowledgments

Grateful thanks are due to the women who completed the survey and shared their experiences and the NHS staff who supported the CHOICE Study.
The authors would also like to thank the CHOICE Study Team.

References

1. Weeks, A.D., Alfirevic, Z. Induction of labour: first do no harm. The Lancet. 2022. 400 (10364): 1656-1657 DOI:https:/doi.org/10.1016/S0140-6736(22)02082-7
2. NMPA Project Team. *National Maternity and Perinatal Audit: Clinical Report 2022. Based on births in NHS maternity services in England and Wales between 1 April 2018 and 31 March 2019.* London: RCOG; 2022.
3. Harkness, M., Yuill, C., Cheyne, H, Stock, S, J, McCourt, C. On behalf of the CHOICE Study Consortia. *Induction of labour during the COVID-19 pandemic: a national survey of impact on practice in the UK.* BMC Pregnancy Childbirth 21, 310 (2021). https://doi.org/10.1186/s12884-021-03781-x
4. National Institute for Clinical Excellence NICE. *Inducing Labour. Clinical Guideline {CG207}.* Published 4th November 2021. At: https://www.nice.org.uk/guidance/ng207
5. Coates, R, Cupples, G, Scamell, A, McCourt, C, Bhide, A. *Women's experiences of outpatient induction of labour with double balloon catheter or prostaglandin pessary: A qualitative study.* Women and Birth. 2021. 34(4):e406-e415. doi: 10.1016/j.wombi.2020.07.006.
6. Coates, R, Cupples, G, Foya V, McCourt C, Scamell M. *Women's experiences of induction of labour: qualitative systematic review and thematic synthesis.* Midwifery, 2018-10. DOI: 10.1016/j.midw.2018.10.013
7. WHO recommendations: intrapartum care for a positive childbirth experience. Geneva: World Health Organization; 2018. Licence: CC BY-NC-SA 3.0 IGO.

For peer review only - http://bmjopen.bmj.com/site/about/guidelines.xhtml

8. Vedeler C, Nilsen ABV, Blix E, Downe S, Eri TS. What women emphasise as important aspects of care in childbirth–an online survey. BJOG 2022: 128; 647-655
9. Bell A,F, Andersson, E. *The birth experience and women's postnatal depression: a systematic review.* Midwifery. 2016. 39:112-123
10. Stock SJ, Bhide A, Richardson H, et al. *Cervical ripening at home or in hospital—prospective cohort study and process evaluation (CHOICE) study: a protocol.* BMJ Open 2021;11:e050452. doi:10.1136/bmjopen-2021-050452
11. Stock SJ, Taylor R, Mairs R, Azaghdani A, Hor K, Smith I, Dundas K, Kissack C, Norman JE, Denison F. *Home cervical ripening with dinoprostone gel in nulliparous women with singleton pregnancies*. Obstet Gynecol. 2014 Aug;124(2 Pt 1):354-360. doi: 10.1097/AOG.0000000000000394
12. Agarwal K, Batra A, Batra A, Dabral A, Aggarwal A. *Evaluation of isosorbide mononitrate for cervical ripening prior to induction of labor for postdated pregnancy in an outpatient setting.* Int J Gynaecol Obstet. 2012 Sep;118(3):205-9. doi: 10.1016/j.ijgo.2012.04.017
13. Reid M, Lorimer K, Norman JE, Bollapragada SS, Norrie J. *The home as an appropriate setting for women undertaking cervical ripening before the induction of labour.* Midwifery. 2011 Feb;27(1):30-5. doi: 10.1016/j.midw.2009.11.003. Epub 2010 Jan 4. PMID: 20045584
14. Henry, A., Madan, A., Reid, R. et al. *Outpatient Foley catheter versus inpatient prostaglandin E2 gel for induction of labour: a randomised trial.* BMC Pregnancy Childbirth. 2013. 13, 25. https://doi.org/10.1186/1471-2393-13-25
15. Hodnett ED, Simmons-Tropea DA. *The Labour Agentry Scale: psychometric properties of an instrument measuring control during childbirth*. Res Nurs Health. 1987;10(5):301-10.
16. Scottish Government. *Maternity care survey 2018: national results.* Scottish Government Publications. 2019. ISBN: 9781787816398. At: www.gov.scot/publications/maternity-care-survey-2018-national-results/pages/11/
17. Cheyne, H, Abhyankar P, Williams B. *Elective induction of labour: The problem of interpretation and communication of risks.* Midwifery. 2012 http://dx.doi.org/10.1016/j.midw.2012.06.009
18. Borrelli SE, Walsh D, Spiby H. *First-time mothers' expectations of the unknown territory of childbirth: Uncertainties, coping strategies and 'going with the flow*. Midwifery. 2018. 63:39-45
19. Downe S, Finlayson K, Oladapo OT, Bonet M, Gülmezoglu AM. *Correction: What matters to women during childbirth: A systematic qualitative review.* PLOS ONE. 2018. 13(5): e0197791. https://doi.org/10.1371/journal.pone.0197791
20. Birthplace. *Perinatal and maternal outcomes by planned place of birth for healthy women with low risk pregnancies: the Birthplace in England national prospective cohort study.* BMJ 2011; 343:d7400. doi: https://doi.org/10.1136/bmj.d7400 (Published 25 November 2011)
21. Scarf VL, Rossiter C, Vedam S, Dahlen HG, Ellwood D, Forster D, Foureur MJ, McLachlan H, Oats J, Sibbritt D, Thornton C, Homer CSE. *Maternal and perinatal outcomes by planned place of birth among women with low-risk pregnancies in high-income countries: A systematic review and meta-analysis.* Midwifery. 2018 Jul;62:240-255. doi: 10.1016/j.midw.2018.03.024. Epub 2018 Apr 3. PMID: 29727829.
22. Overgaard, C., Fenger-Grøn, M., & Sandall, J. *Freestanding midwifery units versus obstetric units: Does the effect of place of birth differ with level of social disadvantage?* BMC Public Health. 2012. 12(1). https://doi.org/10.1186/1471-2458-12-478

For peer review only - http://bmjopen.bmj.com/site/about/guidelines.xhtml

23. Macfarlane, A.J., Rocca-Ihenacho, L., Turner, L. and Roth, C. *Survey of women's experiences of care in a new freestanding midwifery unit in an inner city area of London, England – 1: Methods and women's overall ratings of care.* Midwifery. 2014. 30(9), pp.998-1008.
24. RCM. *RCM warns of midwife exodus as maternity staffing crisis grows.* Media Release. RCM October 2021. At: https://www.rcm.org.uk/media-releases/2021/september/rcm-warns-of-midwife-exodus-as-maternity-staffing-crisis-grows/
25. Gupta JK, Maher A, Stubbs C, Brocklehurst P, Daniels JP, Hardy P. *Synthetic Osmotic Cervical Dilator for Induction of Labor in Comparison to Dinoprostone Vaginal insErt (SOLVE) collaborative group. A randomized trial of synthetic osmotic cervical dilator for induction of labor vs dinoprostone vaginal insert.* Am J Obstet Gynecol MFM. 2022 Jul;4(4):100628. doi: 10.1016/j.ajogmf.2022.100628. Epub 2022 Mar 28. Erratum in: Am J Obstet Gynecol MFM. 2022 Aug 9;:100702. PMID: 35358740.
26. Knight M, Bunch K, Patel R, Shakespeare J, Kotnis R, Kenyon S, Kurinczuk JJ (Eds.). *Saving Lives, Improving Mothers' Care. Core report: Lessons learned to inform maternity care from the UK and Ireland Confidential Enquiries into Maternal Deaths and Morbidity 2018-20.* November 2022
27. Healthcare Safety Investigation Branch. Assessment of risk during the maternity pathway. Independent Report by the Healthcare Safety Investigation Branch. NI-003397. March 2023. At: https://www.hsib.org.uk/investigations-and-reports/assessment-risk-during-maternity-pathway/report/

For peer review only

For peer review only - http://bmjopen.bmj.com/site/about/guidelines.xhtml

Checklist for Reporting Of Survey Studies (CROSS)

Section/topic	Item	Item description	Reported on page #
**Title and abstract**			
Title and abstract	1a	State the word "survey" along with a commonly used term in title or abstract to introduce the study's design.	P1
	1b	Provide an informative summary in the abstract, covering background, objectives, methods, findings/results, interpretation/discussion, and conclusions.	Abstract
**Introduction**			
Background	2	Provide a background about the rationale of study, what has been previously done, and why this survey is needed.	P1
Purpose/aim	3	Identify specific purposes, aims, goals, or objectives of the study.	P1
**Methods**			
Study design	4	Specify the study design in the methods section with a commonly used term (e.g., cross-sectional or longitudinal).	P1,2
Data collection methods	5a	Describe the questionnaire (e.g., number of sections, number of questions, number and names of instruments used).	P2
	5b	Describe all questionnaire instruments that were used in the survey to measure particular concepts. Report target population, reported validity and reliability information, scoring/classification procedure, and reference links (if any).	P2
	5c	Provide information on pretesting of the questionnaire, if performed (in the article or in an online supplement). Report the method of pretesting, number of times questionnaire was pre-tested, number and demographics of participants used for pretesting, and the level of similarity of demographics between pre-testing participants and sample population.	P2
	5d	Questionnaire if possible, should be fully provided (in the article, or as appendices or as an online supplement).	Appendix
Sample characteristics	6a	Describe the study population (i.e., background, locations, eligibility criteria for participant inclusion in survey, exclusion criteria).	P2
	6b	Describe the sampling techniques used (e.g., single stage or multistage sampling, simple random sampling, stratified sampling, cluster sampling, convenience sampling). Specify the locations of sample participants whenever clustered sampling was applied.	P2
	6c	Provide information on sample size, along with details of sample size calculation.	P2
	6d	Describe how representative the sample is of the study population (or target population if possible), particularly for population-based surveys.	P3,4
Survey	7a	Provide information on modes of questionnaire administration, including the type and number of contacts, the location where the survey was conducted (e.g., outpatient	P2

For peer review only

administration		room or by use of online tools, such as SurveyMonkey).	
	7b	Provide information of survey's time frame, such as periods of recruitment, exposure, and follow-up days.	P2
	7c	Provide information on the entry process:	P3
		–>For non-web-based surveys, provide approaches to minimize human error in data entry.	P3
		–>For web-based surveys, provide approaches to prevent "multiple participation" of participants.	P3
Study preparation	8	Describe any preparation process before conducting the survey (e.g., interviewers' training process, advertising the survey).	P2
Ethical considerations	9a	Provide information on ethical approval for the survey if obtained, including informed consent, institutional review board [IRB] approval, Helsinki declaration, and good clinical practice [GCP] declaration (as appropriate).	P3
	9b	Provide information about survey anonymity and confidentiality and describe what mechanisms were used to protect unauthorized access.	P3
Statistical analysis	10a	Describe statistical methods and analytical approach. Report the statistical software that was used for data analysis.	P3
	10b	Report any modification of variables used in the analysis, along with reference (if available).	N/A
	10c	Report details about how missing data was handled. Include rate of missing items, missing data mechanism (i.e., missing completely at random [MCAR], missing at random [MAR] or missing not at random [MNAR]) and methods used to deal with missing data (e.g., multiple imputation).	Throughout findings section
	10d	State how non-response error was addressed.	N/A P12
	10e	For longitudinal surveys, state how loss to follow-up was addressed.	N/A
	10f	Indicate whether any methods such as weighting of items or propensity scores have been used to adjust for non-representativeness of the sample.	N/A
	10g	Describe any sensitivity analysis conducted.	N/A
**Results**			
Respondent characteristics	11a	Report numbers of individuals at each stage of the study. Consider using a flow diagram, if possible.	N/A
	11b	Provide reasons for non-participation at each stage, if possible.	N/A
	11c	Report response rate, present the definition of response rate or the formula used to calculate response rate.	N/A p12

For peer review only

	11d	Provide information to define how unique visitors are determined. Report number of unique visitors along with relevant proportions (e.g., view proportion, participation proportion, completion proportion).	N/A
Descriptive results	12	Provide characteristics of study participants, as well as information on potential confounders and assessed outcomes.	P4
	13a	Give unadjusted estimates and, if applicable, confounder-adjusted estimates along with 95% confidence intervals and p-values.	N/A
Main findings	13b	For multivariable analysis, provide information on the model building process, model fit statistics, and model assumptions (as appropriate).	N/A
	13c	Provide details about any sensitivity analysis performed. If there are considerable amount of missing data, report sensitivity analyses comparing the results of complete cases with that of the imputed dataset (if possible).	N/A
**Discussion**			
Limitations	14	Discuss the limitations of the study, considering sources of potential biases and imprecisions, such as non-representativeness of sample, study design, important uncontrolled confounders.	P12
Interpretations	15	Give a cautious overall interpretation of results, based on potential biases and imprecisions and suggest areas for future research.	P11 & p12
Generalizability	16	Discuss the external validity of the results.	P12 N/A
**Other sections**			
Role of funding source	17	State whether any funding organization has had any roles in the survey's design, implementation, and analysis.	Submssion declaration
Conflict of interest	18	Declare any potential conflict of interest.	Submssion declaration
Acknowledgements	19	Provide names of organizations/persons that are acknowledged along with their contribution to the research.	Submssion declaration

For peer review only
